# Bmp15 Is an Oocyte-Produced Signal Required for Maintenance of the Adult Female Sexual Phenotype in Zebrafish

**DOI:** 10.1371/journal.pgen.1006323

**Published:** 2016-09-19

**Authors:** Daniel B. Dranow, Kevin Hu, April M. Bird, S. Terese Lawry, Melissa T. Adams, Angelica Sanchez, James F. Amatruda, Bruce W. Draper

**Affiliations:** 1 Department of Molecular and Cellular Biology, University of California Davis, Davis, California, United States of America; 2 Departments of Pediatrics and Molecular Biology, The University of Texas Southwestern Medical Center at Dallas, Dallas, Texas, United States of America; University of Pennsylvania School of Medicine, UNITED STATES

## Abstract

Although the zebrafish is a major model organism, how they determine sex is not well understood. In domesticated zebrafish, sex determination appears to be polygenic, being influenced by multiple genetic factors that may vary from strain to strain, and additionally can be influenced by environmental factors. However, the requirement of germ cells for female sex determination is well documented: animals that lack germ cells, or oocytes in particular, develop exclusively as males. Recently, it has been determined that oocytes are also required throughout the adult life of the animal to maintain the differentiated female state. How oocytes control sex differentiation and maintenance of the sexual phenotype is unknown. We therefore generated targeted mutations in genes for two oocyte produced signaling molecules, Bmp15 and Gdf9 and here report a novel role for Bmp15 in maintaining adult female sex differentiation in zebrafish. Females deficient in Bmp15 begin development normally but switch sex during the mid- to late- juvenile stage, and become fertile males. Additionally, by generating mutations in the aromatase *cyp19a1a*, we show that estrogen production is necessary for female development and that the function of Bmp15 in female sex maintenance is likely linked to the regulation of estrogen biosynthesis via promoting the development of estrogen-producing granulosa cells in the oocyte follicle.

## Author Summary

Domesticated zebrafish use a polygenic mechanism for sex determination, where oocytes play a key role in both promoting primary female sex determination and maintenance of female sex in adults. All animals that cannot produce oocytes during the larval stage develop as males and adult females that cease oocyte production readily sex revert to a male phenotype. These results have led to the hypothesis that oocytes produce a signal that instructs the somatic gonad to develop as an ovary and to suppress testis development. In this report we present evidence that the oocyte-produced signaling protein Bmp15 is required for females to maintain their sexual phenotype, but plays no role in primary female sex determination. Specifically, zebrafish *bmp15* mutants can initially develop as females, but during the juvenile stage they sex revert to fertile males. In addition, we show that the ovary-expressed estrogen- producing enzyme, Cyp19a1a aromatase, is a key somatic gonad-produced protein that promotes female development. Our results define for the first time, specific genetic components of the mechanism by which zebrafish promote and maintain their sex, and may also provide insight into the mechanism(s) that allows 2% of all teleost species to reverse their sex as adults.

## Introduction

Sex determination is the process by which an animal determines whether it will develop as a male or a female. In vertebrates, sex determination is generally divided into two phases. First, during primary sex determination, the primordial bipotential gonad commits to either an ovary or a testis developmental fate. Primary sex determination can be regulated by either a master regulator, such as the Y chromosome-linked Sry transcription factor in mammals, by defined environmental conditions, such as temperature in certain reptiles, or by a more complex polygenic mechanism. Once the gonad has committed to a particular fate, it begins to produce sex-specific hormones that will drive the process of secondary sex determination in which the somatic tissues of the organism differentiate with sex-specific characteristics.

Though in most animals the primary and secondary sex determination mechanisms are considered to lead to a stably differentiated sexual fate, in some animals, sex can be reversed. For example, in teleost fish, an estimated 2% of species reverse their sex at some point during their life [1]. However, even in species that do not normally switch sex during their adult life cycles, such as mammals, there is evidence that sex differentiation of the gonad is not irreversible once established, but instead it requires constant maintenance via sex-specific transcription factors that function to maintain sex-appropriate gene expression. For example, in mice the male-specific transcription factor Dmrt1 is expressed in both germ cells and somatic gonad cells of the testis and conditional deletion of the *Dmrt1* gene in postnatal testes results in a downregulation of testis-specific genes and an upregulation of ovary-specific genes [2]. Thus, it is important to understand the mechanism that allows animals to maintain a stable sexual fate, but at the same time retain enough flexibility in sex determination and differentiation as to allow for functional sex reversal mechanisms to evolve when this trait is advantageous.

Primary sex determination in wild zebrafish appears to be regulated by a ZZ/ZW genetic system, though the gene(s) involved has not been identified [3]. By contrast, several studies have concluded that the most commonly used domesticated zebrafish strains lack any major genetic sex determinant, and instead rely on a polygenic system that is strain dependent (For review, see [4–7]). However, even in wild strains where the W chromosome is necessary for female primary sex determination, it is not absolutely correlated with female development, as a fraction of ZW individuals develop as males [3]. These results show that while a dominant sex-determining chromosome can strongly influence the outcome of sex determination in zebrafish, it is not an absolute prerequisite. This is also true in medaka fish, which use an XX/XY system where a Y-specific duplicate of the *dmrt1* ortholog, called *dmY*, serves as the male sex-determining gene [8–10]. However, under certain conditions, XX animals can develop as males [11–13]. These results argue that while the genetic sex determining systems in these species may be important for maintaining balanced sex ratios under normal circumstances, they are dispensable under certain conditions. Thus, in some species of fish, systems other than dominant sex-determining loci can influence or override a genetic sex-determining strategy.

Zebrafish are classified as juvenile hermaphrodites as all fish initially produce early-stage oocytes between 10–25 days post fertilization (dpf), regardless of their eventual sex [14]. During this period of development somatic gonad cells express a combination of genes that are associated with either testis or ovary development, suggesting that the gonad is bipotential [15]. After primary sex determination, animals that will become male lose oocytes by apoptosis while the oocytes in females continue to mature [16]. Previous studies have demonstrated that germ cells are indispensable for female development as zebrafish that develop without germ cells, or oocytes in particular, become phenotypic males [17–19]. Oocytes are therefore required for primary female sex determination. In addition, recent work from our lab has demonstrated that oocytes are also required throughout adult life to maintain the female sex [20]. These data have led to the hypothesis that oocytes produce a signal(s) that functions to stabilize female-specific gene expression within early somatic gonad cells during the primary sex determination phase of development as well as to maintain the female differentiated state in adults. However, this signal(s) has not been identified.

In vertebrates, bidirectional paracrine signaling between the oocyte and the surrounding granulosa cells is critical for the proper development and function of the follicle. Oocytes receive signals and nutrients from follicle cells throughout their development while the oocyte in turn signals to surrounding follicle cells to regulate their function. For example, the oocyte is deficient in producing key enzymes necessary for glycolysis and cholesterol biosynthesis, and instead produces signals that upregulate these pathways in granulosa cells, which in turn provide these essential compounds to the oocyte via gap junctions [21]. Two of these oocyte-produced signals that regulate granulosa cell development are Growth differentiation factor 9 (Gdf9) and Bone morphogenetic protein 15 (Bmp15), which are closely related members of the TGF-β superfamily of signaling molecules and are expressed primarily by the oocyte in mice and zebrafish [22–26], but their functions have only been extensively studied in mammals [27].

In both mammals and fish, granulosa cells are known (mammals) or are likely to be (fish) the primary source of circulating estrogen, the key hormone involved in female secondary sex determination, and in mice there is evidence that loss of Gdf9 results in decreased *Cyp19a1* expression, thus linking oocyte signaling to potential defects in sexual development [28]. Importantly, pharmacological inhibition of estrogen production in fish causes masculinization [29]. Estrogens are produced from androgen precursors by an enzyme complex, a component of which is encoded by the cytochrome P450 Family member, Cyp19a1 aromatase, which catalyzes the final step of estrogen biosynthesis. Mammalian *Cyp19a1* is expressed most highly in the granulosa cells of the oocyte follicle in the ovary and at significantly lower levels in Leydig cells of the testis [30]. Teleost fish have two genes that encode a Cyp19a1 aromatase, called *cyp19a1a* and *cyp19a1b*, which are products of the teleost-specific whole genome duplication event. In mammals, Cyp19a1 is expressed in both the ovary and brain, whereas in zebrafish the expression of *cyp19a1a* appears to be restricted to the ovary, and *cyp19a1b* is most highly expressed in the brain [31]. Therefore, *cyp19a1a* is likely the key gene that regulates zebrafish female sexual development.

In the present study, we test whether the oocyte-expressed signaling molecules Gdf9 and Bmp15 have any role in female sex determination or maintenance of female sex differentiation in zebrafish. We find that *bmp15* mutant females initially have normal development, but during the juvenile stage, oocytes are degraded after they arrest at early stages, and the premeiotic germ cells switch to a spermatogenic program as the gonad transforms to a fully functional testis. Consequently, all *bmp15* mutant adults are fertile males. Additionally, we find, by mutational analysis, that the somatic gonad-expressed Cyp19a1a aromatase, an enzyme responsible for estrogen production by the ovary, is required for female sexual development because all mutant animals develop as males. Interestingly, we find that in wild-type ovaries, *cyp19a1a* is initially expressed in theca cells, but not granulosa cells that surround early stage oocytes, but then is upregulated in granulosa cells that surround stage II oocytes. In *bmp15* mutants, by contrast, *cyp19a1a* expression in theca cells is apparently normal, but we do not detect its expression in granulosa cells. Together, these results argue that *bmp15* functions to maintain female sex determination, in part by promoting *cyp19a1a* expression, and therefore estrogen production, by granulosa cells. To our knowledge, this is the first instance in a vertebrate of the identification of a germ cell–derived signal that is required for maintaining a specific sexual phenotype.

## Results

### Oocytes are required to maintain the female sex in adults

**Fig 1:**
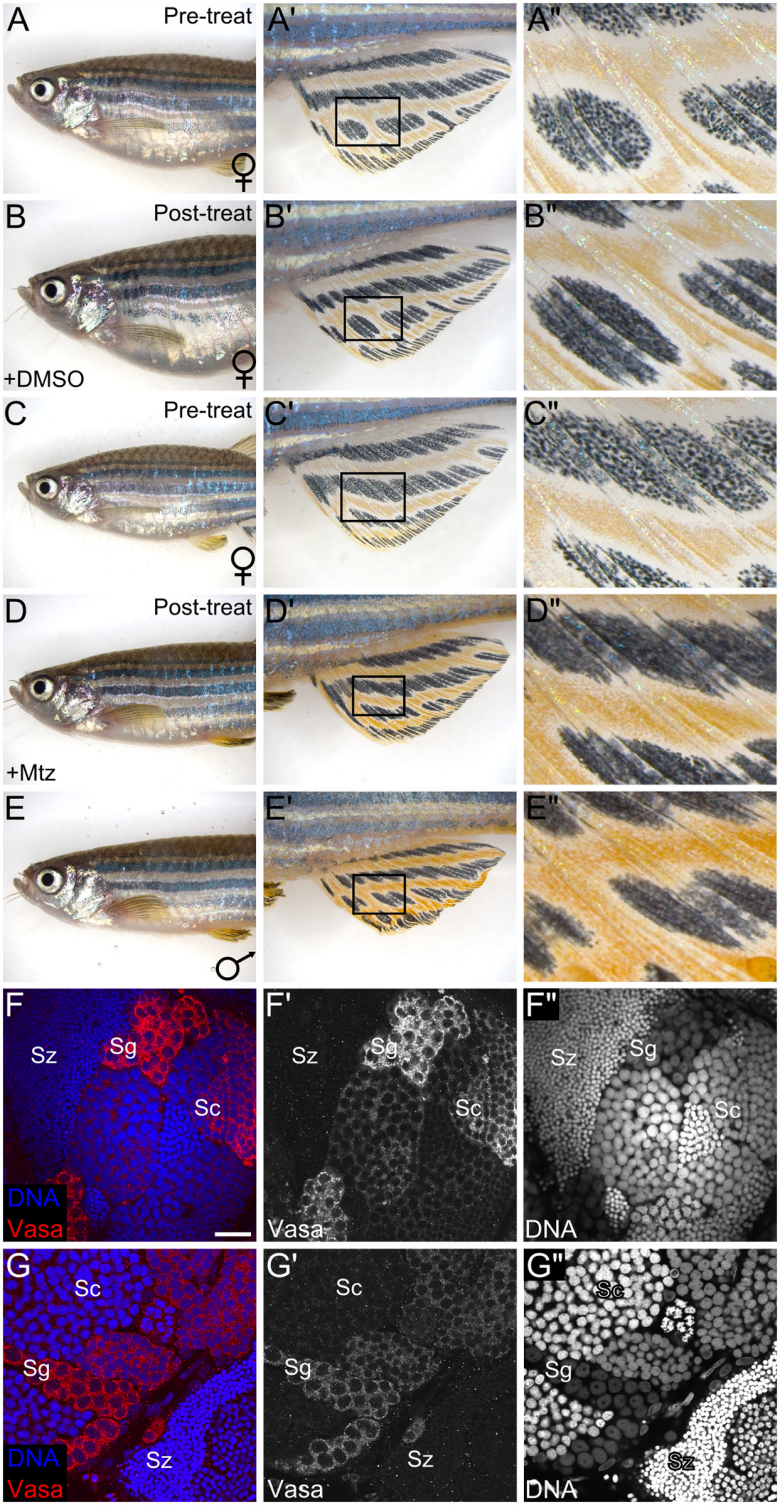
Oocytes are required to maintain the adult female sexual phenotype. (A-A”) Representative *Tg(zp3*:*CFP-NTR)* female prior to control DMSO-treatment and the same fish (B-B”) post-treatment. Female zebrafish (A-B”) have large, egg-filled abdomens and light yellow fin pigmentation. (C-C”) Representative *Tg*(*zp3*:*CFP-NTR*) female prior to Mtz treatment and the same fish (D-D”) two months after Mtz treatment. Note that the post-treatment fish has a slimmer body and darker yellow fin pigmentation characteristic of a DMSO-treated control male (E-E”). (F-G”) Confocal images of testes isolated from a normal male (F-F”) and a representative sex-reverted *Tg(zp3*:*CFP-NTR)* (G-G”) fish. (F, G) Merged images: Vasa protein in red, DNA in blue. (F’, G’) Vasa protein only. (F”, G”) DNA only. (A’-E’) Magnified view of the anal fin. (A”-E”) Magnified view of the regions boxed in A’-E’. Sg, spermatogonia; Sc, spermatocytes; Sz, spermatozoa. Scale bar: 20 μm (F-G”).

Zebrafish that develop without germ cells, or that cannot produce oocytes, are exclusively male [17–19]. In addition, we have previously shown, using two independent methods, that germ cells are also required throughout the adult life to maintain female sex differentiation, as adult females can sex revert to fertile males following the loss of most germ cells [20]. Though these later studies have led to the hypothesis that adult oocytes produce a female-promoting signal, in these experimental paradigms both pre-meiotic and post-meiotic germ cells were depleted, leaving unanswered whether oocytes specifically are required for maintenance of female sex differentiation. To directly test this, we produced transgenic fish in which oocytes only, but not premeiotic germ cells, were targeted for ablation. In transgenic *Tg(zp3*:*CFP-NTR)*^*uc54*^ females, the zona pellucida protein 3 (*zp3*) promoter drives expression of cyan fluorescent protein-nitroreductase fusion protein (CFP-NTR) in only oocytes, rendering them susceptible to apoptosis after addition of the prodrug, metronidazole (Mtz) [32,33] (S1 Fig). Adult 5-month-old *Tg(zp3*:*CFP-NTR)*^*uc54*^ females were treated with Mtz and allowed to recover for two months before being assessed for changes in external phenotypic characteristics and sex-specific behavior, as previously described [20]. We found that following Mtz treatment, 13/21 (62%) treated females appeared phenotypically identical to wild-type males (Fig 1A–1E”). Of those, 9/13 were capable of inducing wild-type females to spawn in mating trials with 2/9 crosses yielding viable offspring (4/933 viable embryos from all crosses). Importantly, regardless of fertility in mating trials, dissection of all fish with a male-like phenotype revealed that 13/13 (100%) had apparently normal testes that contained germ cells representing all stages of spermatogenesis (Fig 1F–1G). Though we do not understand why these animals are sub-fertile, one possibility is that testes derived from sex-reverted ovaries do not make fully functional connections to the cloaca. Nevertheless, these results reveal that, similar to what has been shown previously for primary sex determination [19], oocytes alone are required to maintain female sex differentiation, and thus prevent sex reversal, in adult zebrafish.

### Oocyte-produced Bmp15 is required for maintenance of the female sex in juveniles

The zebrafish follicle consists of an oocyte that is surrounded by a single layer of granulosa cells and an outermost layer of steroid-producing theca cells. Paracrine signaling between oocytes and granulosa cells has been shown to be essential for coordinating the growth of the follicle during the various stages of oogenesis [21]. Two such oocyte-specific paracrine signals are the closely related growth differentiation factor 9 (Gdf9) and bone morphogenetic protein 15 (Bmp15). Both *gdf9* and *bmp15* are expressed in zebrafish oocytes [24,25] (S2 Fig), as early as 22 dpf [34], and are therefore candidates for an oocyte-derived female sex determination and/or maintenance of sex differentiation signal in zebrafish. To test this possibility, we generated frame-shift deletion mutations in the 5’ ends of the *gdf9* and *bmp15* loci using transcription activator-like effector nucleases (TALENs) that are expected to result in complete loss-of-function alleles [35] (S3 Fig; see S1 Table for allele sequences). In mice, *Gdf9* loss-of-function results in oocyte loss and female sterility whereas *Bmp15* mutants have a less severe sub-fertile phenotype, with minor defects in oocyte maturation and ovulation [36]. In zebrafish, homozygous mutants for two different *gdf9*^*-/-*^ null alleles had no discernable phenotype, yielding both fertile adult females and males (S4 Fig and see below), suggesting that *gdf9* does not play an essential role in oocyte maturation or female sex determination and/or maintenance of sex differentiation in zebrafish. By contrast, we found that all *bmp15* mutants were males as adults (S5 Fig). Thus, *bmp15*, but not *gdf9*, appears to be required for female sex determination and/or maintenance of sex differentiation.

The phenotype of *bmp15* mutants could result from a failure of primary sex determination or from a failure to maintain female sex during the juvenile and/or adult stages. To distinguish between these possibilities, we utilized *Tg(ziwi*:*EGFP)*^*uc02*^ transgenic animals that allows us to identify definitive females at 30 dpf based on the level of GFP expression in their gonad [37]: Though there is a range of expression within a given population, it is possible to reliably identify presumptive females by selecting those that have the largest, brightest-GFP+ gonads. By comparison, presumptive males have significantly smaller gonads and therefore less overall GFP expression (S6 Fig). Similar transgenics have been previously used to identify males and females at this stage of development [38]. To produce homozygous *bmp15* mutants, we either incrossed *bmp15*^*+/-*^; *Tg(ziwi*:*EGFP)*^*uc02*^ animals or crossed *bmp15*^*+/-*^; *Tg(ziwi*:*EGFP)*^*uc02*^ females with *bmp15*^*-/-*^; *Tg(ziwi*:*EGFP)*^*uc02*^ males, and then selected the females based on GFP expression levels at 30 dpf. We then genotyped these fish and determined their sex at five months of age. As expected, nearly all wild-type (*bmp15*^*+/+*^ and *bmp15*^*+/-*^) fish were female (n = 193/197; 98%), validating the accuracy of our selection strategy. Conversely, we found that all *bmp15* homozygous mutant fish were phenotypically male (n = 165/165; 100%). Furthermore, these *bmp15*^*-/-*^ mutant adults were capable of inducing wild-type females to spawn in mating trials and produced viable offspring.

### Oogenesis is arrested and oocytes degenerate in *bmp15* mutants during juvenile stages

**Fig 2:**
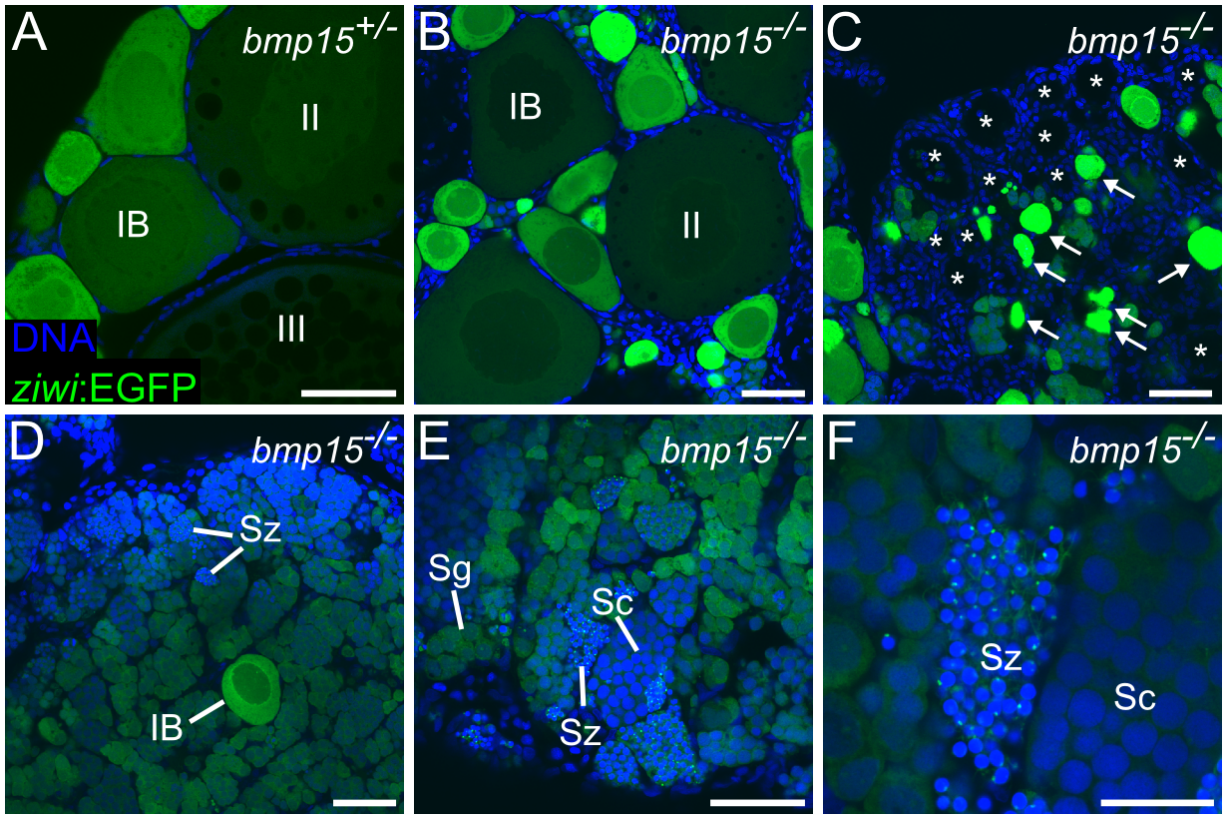
*bmp15*^*-/-*^ females lose oocytes and then produce sperm. (A-F) *ziwi*:*EGFP* expression in gonads of *bmp15*^+/-^ and *bmp15*^-/-^ females at 80 dpf. (A) Representative *bmp15*^+/-^ ovary with stage IB, stage II, and stage III oocytes indicated. (B-F) Representative regions of a single *bmp15*^-/-^ gonad depicting stage IB and stage II oocytes (B), evidence of oocyte degradation and loss (C), spermatogonia surrounding a single stage IB oocyte (D), and a region containing all stages of spermatogenesis (E), including mature sperm (magnified in F). *ziwi*:*EGFP* in green, DNA in blue. Arrows indicate degrading oocytes and asterisks indicate empty follicles. Sg, spermatogonia; Sc, spermatocytes; Sz, spermatozoa. Scale bars: 50 μm (A-E), 20 μm (F).

We next analyzed the sex reversion process more closely in *bmp15* mutants. We found that at 40 dpf, wild-type and mutant ovaries were indistinguishable from each other and contained oocytes of similar stages (S7 Fig; [39]), consistent with the above results that indicated Bmp15 has no role in either primary or secondary female sex determination and differentiation. However, over the next 15–45 days of development (55–85 dpf), we found a progressive loss of oocytes and gain of germ cells that appeared to have initiated a spermatogenic program. Fig 2 shows representative images of the *bmp15* mutant gonad phenotype (Fig 2B–2F) in comparison to a wild-type ovary (Fig 2A) at 80 dpf, showing regions of mutant ovaries that contain mostly oocytes (Fig 2B), a mixture of degrading oocytes and empty follicles (Fig 2C), a mixture of oocytes and spermatogenic germ cells (Fig 2D), and a region that has entirely converted to a testis morphology (Fig 2E), which includes mature sperm (Fig 2F). Finally, by 3 months of age, all *bmp15* mutant fish that were originally selected as female were indistinguishable from wild-type males as assayed by fin pigmentation (S5 Fig).

Although the results so far suggest that *bmp15*, but not *gdf9*, is the key oocyte-produced signal responsible for maintenance of female sex during larval stages, it was still possible that *gdf9* played a minor role that was not revealed by single mutant analysis. We therefore performed double mutant analysis of *bmp15* and *gdf9*. Similar to above, we selected females at 30 dpf based on the expression levels of the *Tg(ziwi;EGFP)* transgene and then analyzed their gonads at 70 dpf. We found that the ovaries of *bmp*^+/-^; *gdf9*^+/-^ and *bmp15*^*+/-*^; *gdf9*^*-/-*^ were indistinguishable from each other and from *bmp15*^*+/-*^; *gdf9*^*+/+*^ ovaries. Notably, all ovaries from these genotypes contained stage III oocytes (compare S8A and S8E Fig, and Fig 2A) and as adults these females were fertile and produced viable embryos (a minimum of four females were fertility-tested for each genotype). By contrast, we found that similar to the gonads of *bmp15*^*-/-*^; *gdf9*^*+/+*^ (Fig 2B), the gonads of both *bmp15*^-/-^; *gdf9*^+/-^ and *bmp15*^*-/-*^; *gdf9*^*-/-*^ females contained only stage II and earlier stage oocytes (S8C and S8F Fig; 5/6 and 4/5 respectively) or spermatogenic cells amongst oocytes, a phenotype indicative of female-to-male sex reversion (S8D and S8G Fig; 1/6 and 1/5 respectively). Based on these results we conclude that *gdf9* does not have an evident role in maintenance of female sex differentiation or in oogenesis.

### The Cyp19a1a aromatase is required for female sexual development

**Fig 3:**
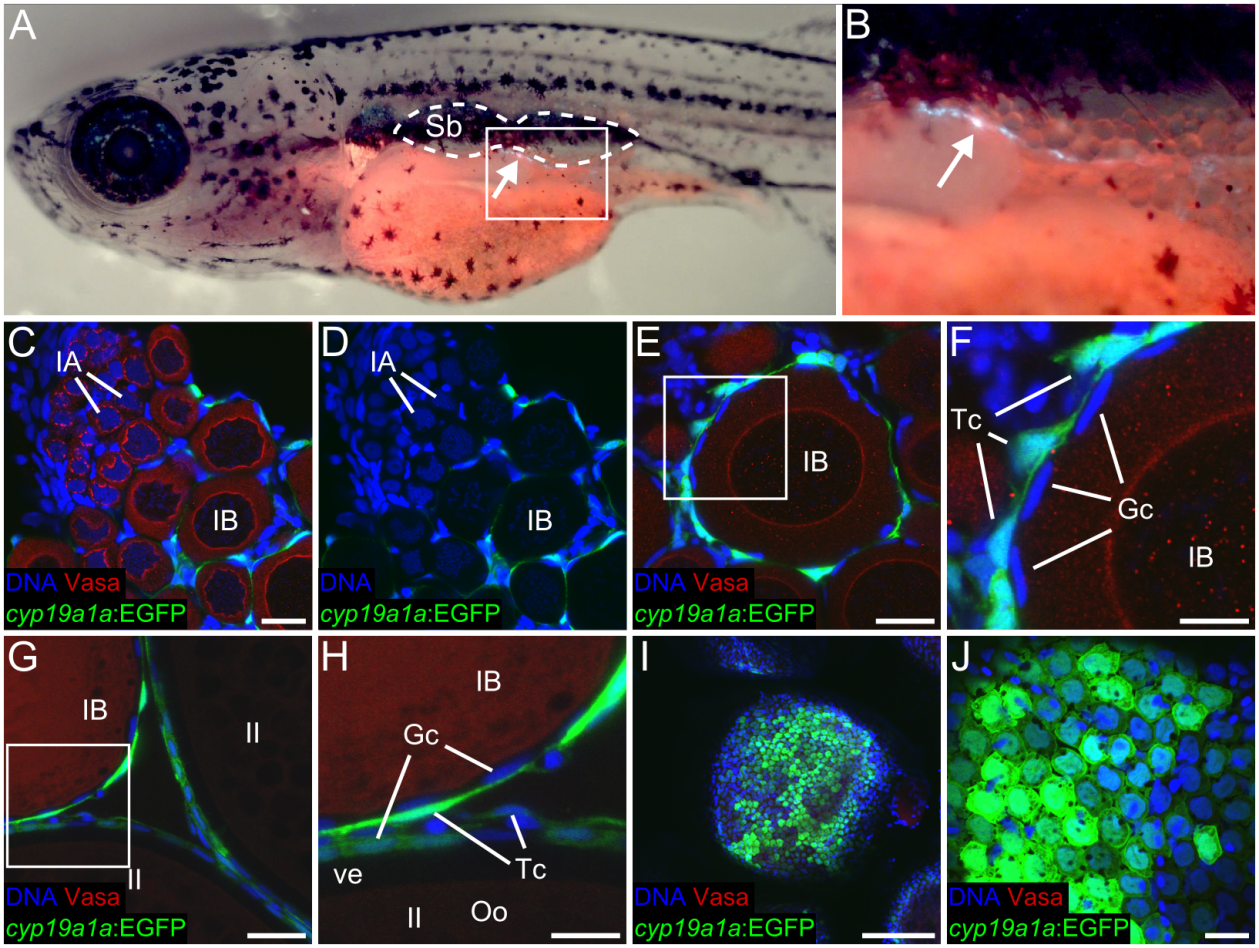
*cyp19a1a*:*EGFP* expression in *TgBAC(cyp19a1a*:*EGFP)*. (A-C) *cyp19a1a*:*EGFP* expression in the gonad at 16 dpf. (A) Anterior body of 16 dpf *TgBAC(cyp19a1a*:*EGFP)* fish showing GFP-expressing somatic gonad cells (arrow) located below the swim bladder (outlined). (B) Magnified view of the region boxed in (A). (C-F) *cyp19a1a*:*EGFP* is expressed in theca cells that surround stage IB and later stage oocytes (E-H), but not IA oocytes or premeiotic germ cells (C, D), as observed here in a 40 dpf ovary. (F) Magnified view of the region boxed in (E). (G-J) *cyp19a1a*: *EGFP* is expressed in both theca and granulosa cells that surround stage II and later stage oocytes, as observed here in a 60 dpf ovary. (H) magnified view of region boxed in (G). (I, J) low and high magnification views of *cyp19a1a*:*EGFP* expression in granulosa cells that are on the surface of a stage III oocyte. In all images, *cyp19a1a*:*EGFP* is green, Vasa protein is red and DNA is blue. Sb, swim bladder; IA, stage IA oocytes; IB, stage IB oocyte; II, stage II oocyte; Tc, theca cell; Gc, granulosa cell; Oo, oocyte; ve, vitelline envelope. Scale bars: 20 μm in C (for C, D, E, G, J), 10 μm in F (for F and H), 150 μm (I).

The zebrafish genome contains two ohnologs of mammalian aromatase *Cyp19a1*, called *cyp19a1a* and *cyp19a1b*, which are expressed in the ovary and brain, respectively. Therefore, we reasoned that *cyp19a1a* is most likely involved in female sexual development. To test this hypothesis we first analyzed the expression of *cyp19a1a* in the ovary. Although *cyp19a1a* has been previously shown to be expressed most highly in the ovary, the dynamics of its expression have not been determined at high resolution in zebrafish [15]. We therefore produced a stable *cyp19a1a*:*EGFP* reporter transgenic line, called *TgBAC(cyp19a1a*:*EGFP)*^*uc44*^, using bacterial artificial chromosome (BAC) transgenesis [40]. Consistent with previous reports, we found that *TgBAC(cyp19a1a*:*EGFP)*^*uc44*^ expression could first be detected in the early gonad beginning at 13 dpf (Fig 3A and 3B show expression at 16 dpf). We also found that it was expressed in theca cells surrounding oocytes from stage IB and older, and in granulosa cells that surround stage II and older oocytes, but not in pre-granulosa cells that surround stage IA oocytes (Fig 3C–3J).

To directly test the necessity of *cyp19a1a* and *cyp19a1b* for female development, we used TALENs to produce loss-of-function mutations in both genes (S3 Fig). Given that pharmacological inhibition of aromatase activity causes all-male development in zebrafish and that *cyp19a1a* RNA is enriched in the ovary but not the testis, we predicted that all *cyp19a1a*^*-/-*^ mutant fish would develop as males. We found that, while the heterozygote population consisted of both males and females (45 males, 49 females), all homozygous mutant animals were fertile males as adults (77 males, 0 females; S9 Fig). By contrast, the sex ratios of *cyp19a1b* mutants were indistinguishable from their wild-type siblings, indicating that *cyp19a1b* does not play a major role, if any, in zebrafish sex determination (males, 15 +/+: 45 +/-: 18 -/-, *x*^*2*^ *p*>0.1; females, 28 +/+: 45 +/-: 25 -/-, *x*^*2*^ *p*>0.5).

**Fig 4:**
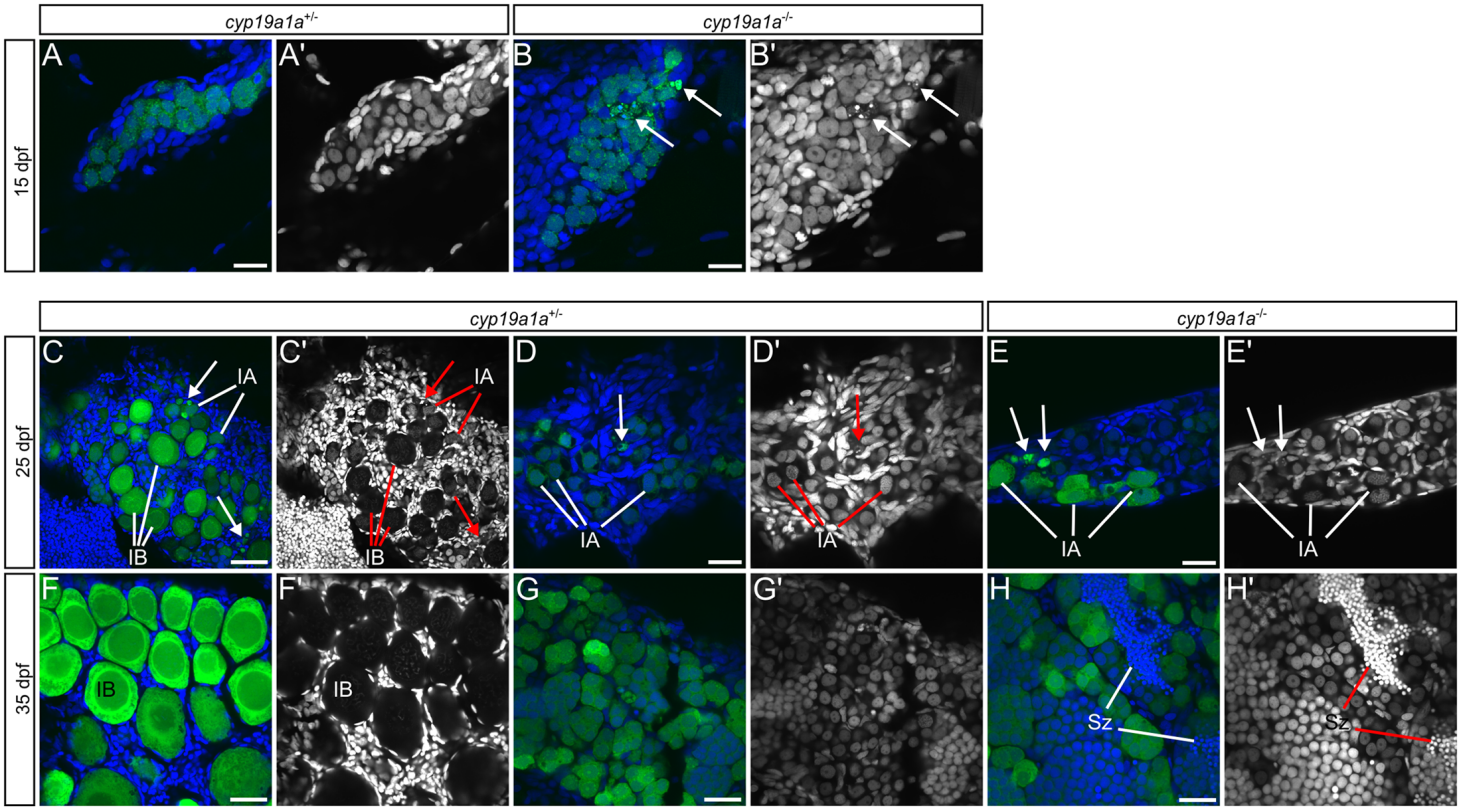
*cyp19a1a* mutant gonads precociously develop into testes. (A-H’) *cyp19a1a* heterozygous (A, C, D, F, G) and mutant (B, E, H) gonads at 15 (A-B’), 25 (C-E’), and 35 (F-H’) dpf. Wild-type presumptive ovary (C) and ovary (F). Wild-type presumptive testis (D) and testis (G). Mutant presumptive testis (E) and testis (H). (A-H) Merged images: *ziwi*:*EGFP* in green, DNA in blue. (A’-H’) DNA only. Arrows indicate degrading germ cells. IA, stage IA oocyte; IB, stage IB oocyte; Sz, spermatozoa Scale bars: 20 μm (A-E), 50 μm (F -H’).

We next determined at what stage *cyp19a1a* was required for female sexual development. During normal development, all zebrafish larvae produce early-stage oocytes between 10–25 dpf, at a time when the somatic gonad is still bipotential [16]. To determine whether Cyp19a1a was required for the production of these early stage oocytes or alternatively only for their maintenance, we examined the gonads of *cyp19a1a* mutants during the bipotential/primary sex determination stage. We found that at 25 dpf, most wild-type and *cyp19a1a* mutant gonads contained early stage oocytes (stage IA), indicating that *cyp19a1a* is not required for this early wave of oocyte production (Fig 4C–4E’). In wild-type animals that will become males, the oocytes that are initially produced will begin to undergo apoptosis around 25 dpf (Fig 4D and 4D’; [16]). Germ cell apoptosis at this stage is not exclusive to males as many oocytes in females also undergo apoptosis shortly after they enter meiosis, similar to what occurs in mice (Fig 4C and 4C’; [41]). However, female and male gonads can be distinguished at 25 dpf based on the presence in females (n = 2/5) (Fig 4C and 4C’) or absence in males (n = 3/5) (Fig 4D and 4D’) of stage IB oocytes. Consistent with *cyp19a1a* mutants developing as males, their gonads at 25 dpf contain fragments of apoptotic germ cells and no stage IB oocytes (n = 3/3) (Fig 4E and 4E’). Importantly, in wild-type gonads, regardless of eventual sex, no oocyte apoptosis was observed at 15 dpf (n = 0/5) (Fig 4A and 4A’). By contrast, in *cyp19a1a* mutants, we found germ cell apoptosis was evident as early as 15 dpf in 83% (n = 5/6) of animals (Fig 4B and 4B’), suggesting that mutant gonads may begin transformation into testes at an earlier time point than do wild type males [16]. Consistent with this idea, by 35 dpf, all *cyp19a1a* mutant gonads contain meiotic spermatocytes (n = 7/7). While mature sperm have not been observed in control 35 dpf testis (n = 0/12), they were observed in a mutant testis (n = 1/7). Based on these observations, we conclude that the first wave of oocyte production in the early gonad does not require Cyp19a1a function, but that Cyp19a1a appears to be required to stabilize a female developmental program and in its absence, the early gonad initiates definitive testis development precociously.

### *bmp15* mutants do not develop *cyp19a1a* (+) granulosa cells

**Fig 5:**
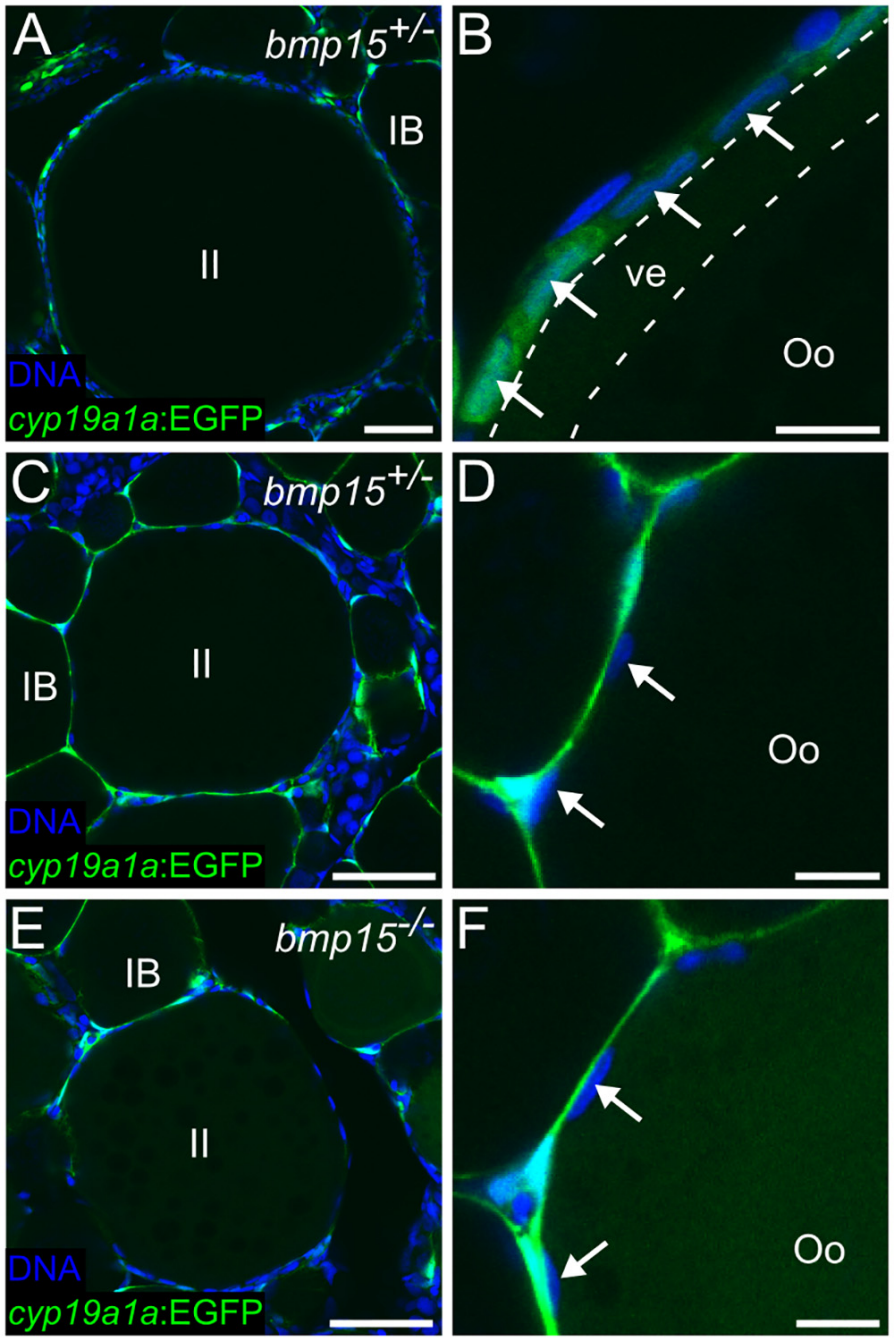
*bmp15*^*-/-*^ oocytes arrest at early stage II and do not develop *cyp19a1a*+ granulosa cells or a vitelline envelope. (A) The maximum sized oocytes observed in 55 dpf *bmp15*^+/-^ ovaries are mid stage II (~280 μm in diameter), while the maximum sized oocytes observed in *bmp15*^-/-^ sibling ovaries are early stage II, (~150 μm in diameter; E). (B) Representative magnified view of a mid stage II oocyte follicle in *bmp15*^+/-^ ovary showing *cyp19a1a*:*EGFP* -expressing granulosa cells (arrows) and a developed vitelline envelope (between dotted lines). (C) Representative early stage II oocyte found in 55 dpf *bmp15*^+/-^ ovaries and magnified view in (D). (F) Representative magnified view of an early stage II oocyte follicle in *TgBAC(cyp19a1a*:*EGFP);bmp15*^-/-^ ovaries showing that while GFP is expressed in theca cells (green), it is not expressed in any granulosa cells, as is the case for similarly staged *bmp15*^+/-^ oocytes (C, D). In all images, *cyp19a1a*:*EGFP* is green and DNA is blue. IB, stage IB oocyte; II, stage II oocyte; Oo, oocyte; ve, vitelline envelope. Arrows indicate granulosa cell nucleus. Scale bars: 100 μm (A, C, E), 50 μm (B, D, F).

The results above indicate that *cyp19a1a* gene function is required for female sexual development and perhaps is the pivotal target of the oocyte-dependent pathway that distinguishes female from male development. It is likely to also be required for maintenance of female sex differentiation and thus a potential downstream target of the Bmp15-dependant female sex maintenance pathway. Specifically, if one function of Bmp15 is to maintain or upregulate *cyp19a1a* expression in ovaries, then loss of Bmp15 signaling may lead to reduced *cyp19a1a*-dependent estrogen production by the somatic gonad, and consequently a failure to maintain female sex differentiation. We therefore asked if there was a defect in the expression of the *TgBAC(cyp19a1a*:*EGFP)*^*uc44*^ transgene in *bmp15* mutants. We found that, similar to wild-type, *bmp15* mutants express *TgBAC(cyp19a1a*:*EGFP)*^*uc44*^ in theca cells surrounding stage IB and later stage oocytes (Fig 5E and 5F). However, we found that although wild-type siblings can produce stage II oocytes in excess of 150 μm in size that have *cyp19a1a*:*EGFP* (+) granulosa cells (Fig 5A and 5B), mutant stage II oocytes appear arrested at a size around 150 μm and *TgBAC(cyp19a1a*:*EGFP)*^*uc44*^ expression could not be detected in any granulosa cells (Fig 5E and 5F). These results strongly suggest that *bmp15* mutant ovaries have reduced or no estrogen-producing granulosa cells, thus linking Bmp15 function with estrogen production.

### Putative Bmp15 receptors are expressed in somatic gonad cells

**Fig 6:**
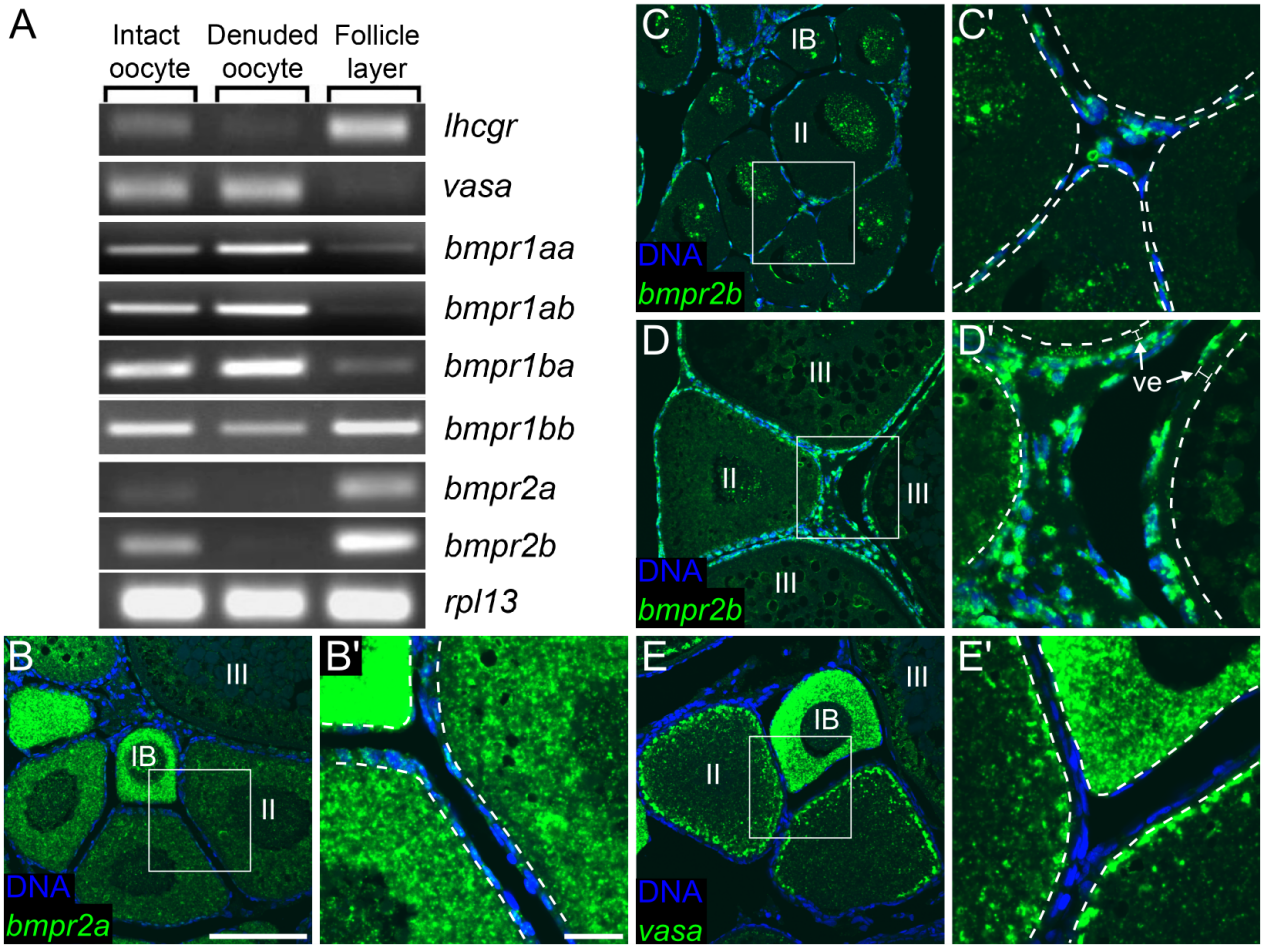
Somatic follicle cells express putative BMP15 receptors. (A) RT-PCR profiling of BMP receptor family members in ovarian tissue. Intact oocyte (consisting of oocyte plus associated follicle cells; left column) were mechanically separated into denuded oocyte (middle column) and follicle cell layer (right column). Reference genes mark follicle cells (*lhcgr*) and oocytes (*vasa*). Expression of the Type I receptor *bmpr1bb* and the Type II receptors *bmpr2a* and *bmpr2b* is selectively enriched in follicle cells. *rpl13* is shown as an amplification control. High resolution fluorescent RNA *in situ* hybridization reveals that *bmpr2a* (green in B) is expressed in early oocytes and in the somatic gonad cells that surround them; *bmpr2b* (green in C, D) is expressed in somatic gonad cells that surround all oocytes; *vasa* (green in E) is only expressed in oocytes (B’-E’ are higher magnification views of areas boxed in B-E, respectively). In all panels DNA in blue. Dotted lines in B’-E’ mark the oocyte membrane; ve, vitelline envelope; IB, II and III denote oocyte stages. Scale bars: 100 μm (B-E), 20 μm (B’-E’).

Our results so far suggest that oocyte-produced Bmp15 regulates the development of somatic gonad cells in the ovary. If so, then the somatic gonad cells should express the appropriate receptors. Bmp proteins function as dimers and bind tetrameric transmembrane Ser-Thr kinase receptors consisting of two Type 1 and two Type 2 subunits. Using cultured human granulosa cells, human BMP15 has been shown to interact most strongly with the type 1 BMP receptor 1B (BMPR1B/Alk6) and the type 2 receptor BMPR2 [42,43]. The zebrafish genome contains four Bmp type 1 receptors (*bmpr1aa*, *ab*, *ba*, *bb*) and two type 2 receptors (*bmpr2a*, *b*). Previous studies have determined that transcripts from all six of these genes can be detected as maternally-provided products in early embryos, indicating that they are all expressed at some point in developing oocytes [44–46]. To determine if any of these can be detected in somatic gonad cells, we first used reverse transcription-polymerase chain reaction (RT-PCR) to compare the expression of these genes in intact stage IV (mature) follicles, in stage IV oocytes that had their somatic cells removed (denuded oocytes) and in isolated somatic cells. We found that while we could detect all of the type 1 receptors in denuded oocytes and in isolated somatic cells, expression of the type 2 receptors appeared to be greatly enriched in the isolated somatic cells (Fig 6A). To further this analysis, we performed fluorescent RNA *in situ* hybridization on sections of paraffin–embedded adult ovaries to examine at high resolution which cells express *bmpr2a* and *bmpr2b*. We found that expression of *bmpr2a* could be detected in stage I and II oocytes, as well as in the somatic cells that surround these and older oocytes (Fig 6B and 6B’). *bmpr2b* expression was detected in apparently all somatic cells, including those surrounding stage II oocytes, but was minimally expressed in oocytes (Fig 6C, 6C’, 6D and 6D’). By contrast, expression of germ-cell specific *vasa* was only detected in germ cells, but not in somatic gonad cells (Fig 6E and 6E’). Together, these data support the hypothesis that oocyte-produced Bmp15 acts on the somatic cells of the follicle to maintain a female-type gene expression profile.

## Discussion

Here we have provided evidence that the oocyte-produced signaling molecule Bmp15 is required for maintenance of the female sexual fate in juvenile zebrafish and that it exerts this function by acting on the somatic gonad cells of the oocyte follicle. We also tested the hypothesis that one of the key female-specific somatic gonad-expressed genes downstream of the Bmp15 oocyte signal is *cyp19a1a*, which encodes a P450 aromatase enzyme responsible for the conversion of androgens to estrogens, and found that *cyp19a1a* is necessary for female sexual development. We found that in the absence of *bmp15* signaling, granulosa cells which surround the oocyte fail to express *cyp19a1a* as they do in wild-type ovaries, providing a possible mechanistic link between an oocyte-produced signal and estrogen production by the somatic gonad. Finally, and in further support of our model, we have found that probable Bmp15 receptors are expressed in somatic follicle cells surrounding all oocytes. These results provide the beginnings of a framework for understanding the importance of germ cell-somatic gonad integrations for sex determination and maintenance of sex differentiation in zebrafish, and perhaps other vertebrates.

### Oocytes are required for female sex determination and maintenance in zebrafish

In both zebrafish and medaka, animals that cannot produce oocytes develop as males, regardless of their genotype [11,17–19], and as we have shown here, oocytes are also required continuously during zebrafish adult life to maintain female sex determination [20]. These and other studies have led to the hypothesis that oocytes produce a signal(s) that functions to stabilize female-specific gene expression in the somatic gonad, thereby promoting an ovarian fate in the early gonad. In both species, there is a correlation between germ cell numbers and subsequent sex: The gonads of eventual females generally have more germ cells than males during the period when primary sex determination occurs [47,48]. If there is a direct correlation between germ cell numbers and the eventual number of oocytes an animal can produce by the end of the sex determination stage, then animals with increased total germ cells will have a higher probability of producing the threshold number of oocytes and oocyte signal(s) required to stabilize female-specific gene expression in the gonad. It is therefore possible that the genetic components of sex determination in wild zebrafish or in their laboratory breed descendants, ultimately control sex by regulating germ cell proliferation during early development. For example, the ZZ/ZW system may promote germ cell proliferation in females (or entry of germ cells into a female-type meiotic program). Nonetheless, in both zebrafish and medaka fish, oocytes play a key role in female gonad development and thus it is essential to identify the signals they produce and how these signals regulate the development and function of the ovary.

### Sex determination vs. maintenance of sex differentiation

In zebrafish, primary sex determination, which determines the sex of the gonad, occurs during the larval stage (5–25 dpf) and secondary sex determination, which involves sex hormone-regulated differentiation of sex-specific soma and behavioral traits, occurs progressively during the juvenile stage (25–90 dpf). Though it was possible that a single oocyte signal was responsible for both sex determination and then subsequent maintenance of the female sexual phenotype, our results clearly demonstrate that Bmp15 is not required for primary female sex determination, as the gonads of *bmp15* mutants can initiate an ovarian developmental program. Instead, our results argue that Bmp15 signaling is required for maintenance of the ovarian fate during the juvenile stage (between 40–90 dpf). Consistent with this finding, *bmp15* expression appears to initiate in follicle stage oocytes (stage IB) whereas current evidence indicates that the signal that influences female primary sex determination must be produced by pre-follicle stage oocytes (stage IA), as these are the only stage oocytes that are present during the early larval stage when sex is likely determined [19]. A corollary of this result is that the oocyte signal(s) that is required for female primary sex determination is not involved in, or at least is not sufficient for, long-term maintenance of female sex differentiation. Thus, at least two independent oocyte-produced signals control two separate aspects of female sexual development. The identity of the signal responsible for female primary sex determination remains to be determined.

Our results provide the first mechanistic insights into how oocytes regulate the maintenance of sex in zebrafish. Our overall hypothesis is that the outcome of the oocyte-somatic gonad interaction is the stabilization of *cyp19a1a* expression and thus estrogen production by the somatic gonad. Consistent with this, we have shown that the somatic gonad expresses probable receptors for Bmp15. In this model, *cyp19a1a* functions as a toggle switch to determine female vs. male development: high *cyp19a1a* will lead to high estrogens and low androgens (female fate), while low or no *cyp19a1a* expression will lead to high androgens and low estrogens (male fate). However, it is unlikely that the sole function of Bmp15 is to regulate *cyp19a1a* expression, nor is it likely that Bmp15 is a direct regulator of *cyp19a1a*. In mice, Gdf9 and Bmp15 are known to influence the development of granulosa cells which surround the oocytes and coordinate their development with that of the oocyte. In the absence of Gdf9 or Bmp15, granulosa cells have reduced expression of the enzymes necessary for glycolysis and cholesterol biosynthesis, the products of which are required by the oocyte, and a decreased rate of proliferation [49,50]. As a result, mutant mice are either sterile (*Gdf9*) or sub-fertile (*Bmp15*; Reviewed by [21]). Though there is evidence that Gdf9 has a positive influence on the expression of the Cyp19a1 aromatase, a direct link has not been established [28]. Finally, in contrast to mice, loss of *Bmp15* in sheep leads to complete sterility, indicating that there are species-specific differences in the relative contributions that each of these proteins have in regulating granulosa cell development and function [51].

Both Gdf9 and Bmp15 orthologs had previously been identified in zebrafish and, as in mammals, were shown to be expressed in oocytes [24,25]; our results), though unlike mammals, the role of these proteins in regulating granulosa cell development and function have not been investigated. Given the critical role of Gdf9 in mammalian follicle development, we were surprised to find that complete loss of Gdf9 function in zebrafish resulted in no detectable phenotype, even when only one functional copy of *bmp15* was present (i.e. *bmp15*^+/-^; *gdf9*^-/-^ animals). It therefore remains to be determined what role, if any, Gdf9 plays in the zebrafish ovary. By contrast, we showed that *bmp15* mutants have a fully penetrant phenotype that resembles that of mouse *Gdf9* mutants: Oocytes arrest development at an early stage and then degrade. However, an obvious difference between the two is that unlike mouse *Gdf9* mutants, zebrafish *bmp15* mutant females also undergo sex-reversion during juvenile development. Given the similarities between the mouse and zebrafish oocyte arrest phenotypes, and the observation that this phenotype appears to precede overt sex reversal in mutants, we favor a model where the primary role of Bmp15 is to regulate somatic gonad development and/or function and when this signal is absent, oocytes arrest and degenerate. In this model, sex reversal is then a secondary consequence of oocyte loss, in line with our previous results [20].

Our model is that the oocyte signal(s) regulate female sexual development by stabilizing the expression of the Cyp19a1a aromatase, and thus the production of estrogen. A key difference between ovarian-produced estrogen in mammals and zebrafish is that in mammals, Cyp19a1 expression, and therefore estrogen production, is limited to granulosa cells, whereas in zebrafish, Cyp19a1a is expressed first in theca cells, and then in granulosa cells that surround stage II and more advanced oocytes (reviewed by [52]; this paper). Thus, unlike mammals that require the coordinated function of two ovarian cell types to produce estrogen, the early zebrafish ovary appears capable of producing sufficient levels of estrogen to stabilize primary female sex determination with only theca cells. Theca and granulosa cell expression of *cyp19a1* is not unique to zebrafish as it has also been reported in the distantly related medaka fish and therefore may be widely conserved in teleost fish [53].

Implicit in this model is that somatic gonad cells express the appropriate Bmp15 receptors. Experiments with human BMP15 have identified the type 1 BMP receptor, BMPR1B and the type 2 receptor, BMPR2, as the probable receptors for BMP15 [42,43]. The zebrafish genome contains four genes encoding Bmp type 1 receptors and two genes encoding Bmp type 2 receptors. Using a combination of RT-PCR and high resolution fluorescent RNA *in situ* hybridization we found that expression of all six genes can be detected in somatic gonad cells, thus supporting our model. Analysis of receptor-specific knockouts will be needed to determine in zebrafish which receptor combinations are necessary for Bmp15 signal transduction in the gonad.

### Why do *bmp15* mutants sex-revert?

The observation that *bmp15* mutants can initially develop as female argues that Bmp15 signaling is not required for the initial production of estrogen during the primary sex determining phase, which is consistent with our observation that in *bmp15* mutants, *cyp19a1a* expression in theca cells appears to be normal during the late larval stage. This indicates that theca cells must be capable of producing sufficient levels of estrogen for definitive female sex differentiation and maintenance through the mid-juvenile stage (~55 dpf). Around 50 dpf, wild-type, but not *bmp15* mutants, upregulate *cyp19a1a* in granulosa cells surrounding oocytes which have reached mid-stage II of oogenesis. While it is possible that Bmp15 signaling directly regulates *cyp19a1a* in granulosa cells, it is more likely that this regulation is indirect. For example, in wild type ovaries, *cyp19a1a*(+) granulosa cells first appear around mid-stage II oocytes (~280 μm in diameter) but *bmp15* mutant oocytes arrest development as early stage II oocytes (~150 μm in diameter). Thus the lack of *cyp19a1a*-expressing granulosa cells in *bmp15* mutants may simply be a secondary consequence of the failure of mutant oocytes to progress beyond this early stage, and that mid-stage II oocytes produce an additional signal that more directly regulates *cyp19a1a*. Finally, Bmp15 may instead negatively regulate the expression of genes like *dmrt1*, that encodes a transcription factor which, in mice, is continually required in adult males to both directly promote the expression of male-specific genes and repress the expression of female-specific genes [2]. To distinguish between these possibilities, it will be necessary to determine with high temporal resolution how loss of Bmp15 signaling affects gene expression in the ovary, and in particular, the granulosa cells.

### The role of estrogen synthesis in zebrafish primary sex determination

A central tenet of our model is that one of the critical final steps in the female sex differentiation pathway is the conversion of androgens to estrogen. The gonads of both males and females normally produce androgens, but only the female gonad expresses high levels of *cyp19a1a* that converts androgens to estrogens. Importantly, *cyp19a1a* is first expressed in a subset of somatic gonad cells in the early gonad [19]. Previous studies using pharmacological aromatase inhibitors in a variety of fish species, including zebrafish, have shown that aromatase activity is required for female development [29,54,55]. However the zebrafish genome contains two ohnologs of Cyp19a1 that have subfunctionalized to be expressed in the brain (*cyp19a1b*) and the ovary (*cyp19a1a*) [31,56]. It was therefore an open question whether one or both of these enzymes played a role in female sexual development. By producing loss-of-function mutations in both genes, we have shown that only the ovarian ohnolog (*cyp19a1a)* is necessary for female sexual development as we have not identified any defects in female development or behavior in *cyp19a1b* mutant animals. Though it is not known at what level Cyp19a1a functions in the zebrafish female sex determination and/or differentiation pathway, in turtles, which have temperature dependent sex determination, Cyp19a1 appears to be one of the key temperature-dependent genes atop the sex determination pathway as it is highly expressed at only the female-specific temperature during the temperature sensitive period [57,58]. Thus, modulation of estrogen levels via the regulation of Cyp19a1 expression is an important sex-determining mechanism in other vertebrate species.

It was known that *cyp19a1a* was expressed in a subset of early somatic gonad cells, when all gonads are producing early stage oocytes (10–25 dpf). During this period, *cyp19a1a*-expressing somatic gonad cells are intermingled with *amh*-expressing cells [15], a gene that will later be expressed most highly in Sertoli cells of the testis. It was therefore possible that the initial wave of oocyte production was dependent on estrogen produced by these early *cyp19a1a*-expressing cells. However, we found that the early gonads of most *cyp19a1a* mutants contained early-stage oocytes like their wild-type siblings, arguing that the initial production of oocytes is either not dependent on estrogen, or that an additional *cyp19a1a*-independent source of estrogen is present at these early stages. Nevertheless, our results argue that *cyp19a1a* has a role during this early phase of gonad development. Specifically, wild-type individuals that are committed to male development can be distinguished based on the absence of stage IB oocytes starting around 25 dpf, but mature sperm are not evident until sometime after 35 dpf. By contrast we have observed mature sperm in *cyp19a1a* mutants as early as 35 dpf. These results argue that definitive male sex differentiation occurs precociously in *cyp19a1a* mutants and that one role of *cyp19a1a* is to extend the time during which the gonad remains uncommitted to a particular fate. Finally, though we favor the model that Cyp19a1a is the rate-limiting step in estrogen biosynthesis during the larval stage when sex is being determined, it is also possible that the correct androgen precursor that is required for estrogen biosynthesis by Cyp19a1a is only produced once fish have committed to the female fate.

### Role of cyp19a1a in testis development

Finally, in mice, males that are mutant for *Cyp19a1* are initially fertile, but over time become sterile [59]. By contrast, we have not detected *cyp19a1a* expression in zebrafish testes, and our analysis of *cyp19a1a* mutants shows that Cyp19a1a is not necessary for male fertility in zebrafish. These results are also consistent with a previous study that found no change in zebrafish sperm production or quality following pharmacological inhibition of aromatase activity [60]. However, it is possible that the normal fertility of *cyp19a1a* mutant males is due to estrogen production by the *cyp19a1b* (brain) aromatase. Production and analysis of *cyp19a1a*; *cyp19a1b* double mutants will be necessary to resolve this issue.

### Conclusion

Though several chromosomal regions have been identified that potentially influence sex determination in zebrafish, the causative genes within these regions have not been identified. Here we have identified two genes, *cyp19a1a* and *bmp15*, that are required for normal female sexual development and maintenance of the female differentiated state. These genes thus establish the framework of a genetic pathway required for female sexual development and maintenance in zebrafish that is likely conserved in other fish species. Though we have shown that *bmp15* is required for the maintenance of the female sex in juvenile zebrafish, the identity of the oocyte-produced signal that influences female primary sex determination remains to be identified.

## Materials and Methods

### Fish strains

The wild-type strain *AB was used in the production of transgenic and mutants lines and in mating trials. Zebrafish were maintained as described [61]. The following alleles were used for these studies: *bmp15*^*uc31*^, *gdf9*^*uc23*^, *gdf9*^*uc34*^, *cyp19a1a*^*uc38*^, *cyp19a1a*^*uc43*^, *cyp19a1b*^*uc39*^, *cyp19a1b*^*uc40*^(see S1 Table for allele sequences). The following transgenic lines were used: *Tg(zp3*:*CFP-NTR)*^*uc54*^, *Tg(ziwi*:*EGFP)*^*uc02*^, *TgBAC(cyp19a1a*:*EGFP)*^*uc44*^.

### Plasmid construction and generation of transgenic fish

The *zpc0*.*5* (*zp3*) promoter [32] was amplified from genomic DNA using primers (*attB* sequences underlined) fwd: GGGGACAACTTTGTATAGAAAAGTTGAAAATCCCCATGACATGCTGC and rev: GGGGACTGCTTTTTTGTACAAACTTGATTGCCTGCTGACTAATTAAACC and used in Gateway recombination-based cloning with pDONRP4-P1R (Invitrogen) to produce 5’ entry vector p5E-*zp3*. An LR reaction was performed using p5E-*zp3*, pME-*CFP-NTR* [20,33], and pTolDestR4-R2pA [62] to produce pBD557. To generate transgenic animals, *AB embryos were co-injected at the one-cell stage with 5 picoliters of Tol2 transposase mRNA, prepared as described in [63], and pBD557 plasmid DNA at 5 ng/μl each. Germ-line transgenic founders were identified by screening for CFP expression in germ cells between 25 and 35 days post fertilization using a Leica MZ16 fluorescent stereomicroscope. A single founder was outcrossed to *AB to produce the *Tg(zp3*:*CFP-NTR)*^*uc54*^ transgenic line used in this study.

### Metronidazole treatment

For ablation experiments, metronidazole (Mtz; Sigma M1547) was dissolved in fish water as previously described [20]. Fish were treated with 5 mM Mtz 6 times for a total of 120 hours. The duration of a single treatment ranged between 18–24 hours and fish were returned to system water for recovery for 24–30 hours between treatments. For controls, 5-month old Tg(*zp3*:*CFP-NTR*)^*uc54*^ females were treated in system water containing only 0.8% DMSO and 0.2% clove oil working solution. During all treatments, fish were protected from light. Two months following treatment, fish were photographed, euthanized, and processed for immunohistochemistry.

### TALEN design and construction

TALENs were designed to target the first coding exons of *bmp15*, *gdf9*, *cyp19a1a* and *cyp19a1b* and were assembled using The Golden Gate cloning method [64] as described [35] using the Golden Gate TALEN and TAL Effector Kit (Addgene) and the backbone plasmids pCS2TAL3DD and pCS2TAL3RR. S1 Table lists the DNA sequences that were targeted by each TALEN pair.

### Generation and identification of mutant fish

TALEN RNAs were synthesized by *in vitro* transcription using the mMESSAGE mMACHINE kit (Ambion). TALEN pairs were then co-injected at the one-cell stage at 50–100 pg each TALEN/embryo. Founders were identified by screening sperm DNA by high resolution melt (HRM) analysis [35], using Light Scanner Master Mix (BioFire Defense), a CFX-96 real-time PCR machine and Precision Melt Analysis software (BioRad). Primer sequences for the indicated amplicons used in HRM analysis were as follows, with wild-type amplicon size in parentheses: *bmp15*, fwd AGCCTTTCAGGTGGCACTCG and rev GGGAGAAAGTGTTTTTCAGTGG (374 bp); *gdf9*, fwd TGCCCCTTCAACATGGTAAT and rev AGCGCACATATCTGGAGTCA (111 bp); *cyp19a1a*, fwd ACTGAAAGGGCTCAGGACAA and rev TCCAACTGACCTGGAATGTG (122 bp). After initial identification, subsequent genotyping of offspring was performed by PCR followed by visualization on a 2% agarose gel using primers: *bmp15*, fwd AGCCTTTCAGGTGGCACTCG, rev GGGAGAAAGTGTTTTTCAGTGG (374 bp) and the same primers as for HRM analysis for *gdf9* and *cyp19a1a*. Allele sequences are listed in S1 Table. All alleles were outcrossed a minimum of three times and the mutant phenotypes have always correlated 100% with the mutant genotype.

### BAC recombineering and transgenesis

Bacterial strains for recombineering were obtained from the National Cancer Institute (NCI)—Frederick Biological Resources Branch. BAC CH211-165A16 (GenBank: BX908722.4) containing the *cyp19a1a* CDS including upstream sequence was obtained from the Children’s Hospital Oakland Research Institute BACPAC Resources Center. BAC recombineering was performed as described [40]. Primers were (BAC sequence homology underlined) *cyp19a1a*_*EGFP*_fwd: GTGTACGTCTGCAGTTCAGAGGCTTTAGACTTGCTTCTTAATCCGTTCTTGCCACCATGGTGAGCAAGGGCGAGGAGCTGTTC and cyp19a1a_frt_KAN_frt_rev: CTGCTCAAGATGAGAGTTTCCTCACCATTGATCCAGACACGCACAATGTCCCGCGTGTAGGCTGGAGCTGCTTC for amplification of the EGFP cassette. For PCR amplification of the EGFP cassette to verify recombination and for sequencing, *cyp19a1a*_fwd: AGAGATGACTTGCACACAGCTGAGG, *cyp19a1a*_rev: GAAGACTAATGATGGTAGATGAACTGTCCC. Primers *cyp19a1a*_fwd and pA_seqR: GAATAGGAACTTCCTGCAGGAATTC were used as sequencing primers with the aforementioned PCR product as template. For PCR genotyping injected male founders by sperm squeeze, *cyp19a1a*_fwd and *EGFP*_rev: AGATGAACTTCAGGGTCAGCTTGC were used.

### Whole-mount RNA in situ hybridization

The *cyp191a1a* probe was that used by Siegfried and Nüsslein-Volhard [18]. *bmp15* and *gdf9* probes were produced by amplifying the second exon and 3’ untranslated regions of each, using primers: *bmp15* fwd, AATCACGACGGACTAGATCTTCA and rev, TTCATTGTCTTTCATCGTGACAT; *gdf9* fwd, TTCTTCATGCAAGACATTTCC and rev, AAGAGAGAGGTTGCCCATGA. PCR products were cloned into pGEM-T Easy vectors (Promega) to produce pBD749 (*bmp15*) and pBD750 (*gdf9*). In situ hybridization was essentially performed as described in [65], with the exception that probe hybridization solutions contained 5% dextran sulfate (GE Health Care). Images were acquired using a Zeiss Axiophot microscope equipped with a Leica DFC 550 digital camera.

### Immunohistochemistry

Anti-Vasa antibody [66] was used at 1:2,000 to label germ cells and immunohistochemistry performed essentially as described by [67]. For *Tg(zp3*:*CFP-NTR)*^*uc54*^, gonads were incubated for 10 min in 10 mg/ml RNase A in 1X PBS/0.5% Triton x-100 at 37°C and then DNA was stained using propidium iodide (Invitrogen P3566) according to the manufacturer's protocol. DAPI was used to label DNA for all other fish lines.

### Fluorescent image acquisition and processing

Fluorescent images were acquired using an Olympus FV1000 laser scanning confocal microscope. Acquired images were manipulated in only a linear manner using the levels, brightness and contrast features in Adobe Photoshop CS5.

### Analysis of Bmpr expression by reverse transcription polymerase chain reaction

Female zebrafish were euthanized using Tricaine and the ovaries were removed. Manual separation of follicle layers from mature oocytes was performed as previously described [25]. Total RNA was isolated from intact follicles, follicle layer only or denuded oocytes only using TRIzol reagent (Thermo Fisher Scientific). Reverse transcription of 150ng of total RNA was performed using the RT^2^ First Strand Kit (Qiagen). PCR amplification was performed using the primers listed in S2 Table for the indicated number of cycles. Products were visualized on a 1% agarose gel using EtBr.

### Fluorescent RNA in situ hybridization on paraffin sections

DNA templates for *bmpr2a* and *bmpr2b* RNA *in situ* probe synthesis were generated by PCR using the following primer sets, which amplify exon 12 of each from genomic DNA (T7 promoter sequence is underlined): *bmpr2a* fwd, TGAAGACCATGAAGGTGCTG and rev, GGCGTAATACGACTCACTATAGGGAAATGGCAGTGTTGTGATGGA; *bmpr2b* fwd, TCCCAAAATGGGCTCTTACC and rev, GGCGTAATACGACTCACTATAGGGAGAGGCTGACAGGTCCAGTG. *vasa* probe was produced as previously described[68]. RNA *in situ* hybridization on 7 μm paraffin sections was preformed essentially as described in [69], except that probes were detected using a fluorescent tyramide reaction as described in [70]. Images were acquired using an Olympus FV1000 laser scanning confocal microscope.

### Ethics statement

The University of California Davis IACUC approved all animals used in this study (protocol #18487), and all animals used were euthanized using the American Veterinary Medical Association-approved method of hypothermal shock.

## Supporting Information

S1 Fig The *zp3* promoter drives expression of CFP-NTR in oocytes but not premeiotic germ cells. (A-C) Expression of *zp3*:*CFP-NTR* in the ovary. (A) Merged image: CFP-NTR in green, DNA in blue. (B) CFP-NTR only. (C) DNA only. IB, stage IB oocyte. Arrow indicates premeiotic germ cell. Scale bar in A (for A-C): 20 μm.
(TIF)

S2 Fig *bmp15* and *gdf9* are expressed in oocytes. (A-D) *In situ* hybridization for *bmp15* and *gdf9* RNA in 40 dpf wild-type gonads. (A) *bmp15* and *gdf9* (C) RNAs are detected in stage IB oocytes but not the smaller, more immature stage IA oocytes or premeiotic germ cells in ovaries. Neither transcript is detected in wild-type adult testes (B, D). Scale bar in A (for A-F): 50 μm.
(TIF)

S3 Fig *bmp15*, *gdf9*, *cyp19a1a*, and *cyp19a1b* mutant alleles. *gdf9* (A), *bmp15* (B), *cyp19a1a* (C), and *cyp19a1b* (D) alleles. Each allele is a predicted loss-of-function mutation. Green indicates wild-type sequence while yellow indicates the predicted frame-shifted sequence. Black triangles indicate TALEN target sites with the size of each deletion bracketed above. Asterisks mark the sites of premature stop codons. The predicted length of the corresponding peptide is indicated on the right. WT, wild type; aa, amino acids.
(TIF)

S4 Fig *gdf9* mutants yield fertile adult females and males. *gdf9* mutants (alleles *uc23*, *uc34*) were raised to adulthood and their sex determined.
(TIF)

S5 Fig *bmp15* mutants are males as adults. (A-A”’) Adult *bmp15*^+/-^ female. (B-B”’) Adult *bmp15*^+/-^ male. (C-C”’) Representative adult *bmp15*^-/-^ male. (A-C) Anterior body. (A’-C’) Magnified view of the anal fin. (A”-C”) Magnified view of the regions boxed in A’-C’. (A”’-C”’) Magnified view of the cloaca. While females (A”’) have a prominent cloaca (arrow), males (B”’, C”’) do not.
(TIF)

S6 Fig EGFP expression in germ cells identifies presumptive males and females. (A, B) EGFP expression in *Tg(ziwi*:*EGFP)* fish at 33 dpf. (A) EGFP expression is lowest in presumptive males but greater the larger gonads of presumptive females (B). Arrows indicate the gonad.
(TIF)

S7 Fig *bmp15*^*-/-*^ mutant gonads are normal at 40 dpf. (A-C) *bmp15*^+/-^ ovaries are indistinguishable from *bmp15*^-/-^ ovaries (D-F). (A, D) Merged images: *ziwi*:*EGFP* in green, DNA in blue. (B, E) *ziwi*:*EGFP* only. (C, F) DNA only. IA, stage IA oocytes; IB, stage IB oocytes. Scale bar in A (for A-F): 50 μm.
(TIF)

S8 Fig *gdf9* does not play a role in sex determination or maintenance. *bmp15; gdf9* double mutant analysis (A-G). *bmp15*^*+/-*^; *gdf9*^*+/-*^ (A, B), *bmp15*^*-/-*^; *gdf9*^*+/-*^ (C, D), *bmp15*^*+/-*^; *gdf9*^*-/-*^ (E), *bmp15*^*-/-*^; *gdf9*^*-/-*^(F, G). *ziwi*:*EGFP* in green, DNA in blue. Arrows indicate degrading oocytes and asterisk indicates empty follicle. IB, II, III indicate oocyte stages. Sg, spermatogonia; Sc, spermatocytes; Sz, spermatozoa. Scale bars: 100 μm in A (for A, C, E, F and G), 25 μm (B), 50 μm (D).
(TIF)

S9 Fig *cyp19a1a* mutants are males as adults. (A-A”‘) Adult *cyp19a1a*^+/-^ female. (B-B”‘) Adult *cyp19a1a*^+/-^ male. (C-C”‘) Representative adult *cyp19a1a*^-/-^ male. (A-C) Anterior body. (A’-C’) Magnified view of the anal fin. (A”-C”) Magnified view of the regions boxed in A’-C’. (A”‘-C’”) Magnified view of the cloaca. While females (A”‘) have a prominent cloaca (arrow), males (B”‘, C”‘) do not.
(TIF)

S1 Table TALEN DNA-binding sites are underlined. Sequence between binding sites is lower case and bold. Sequences are in 5’ to 3’ direction.
(TIF)

S2 Table Sequences of oligonucleotide primers used for the reverse transcription polymerase chain reaction experiment shown in Fig 6. The annealing temperature and numbers of PCR cycles used for each gene-specific primer set are listed.
(TIF)
