# Supplementary figures and images for: Bmp15 Is an Oocyte-Produced Signal Required for Maintenance of the Adult Female Sexual Phenotype in Zebrafish
Source: PLoS Genet. 2016 Sep 19;12(9):e1006323. doi: 10.1371/journal.pgen.1006323 (PMC5028036; doi:10.1371/journal.pgen.1006323)

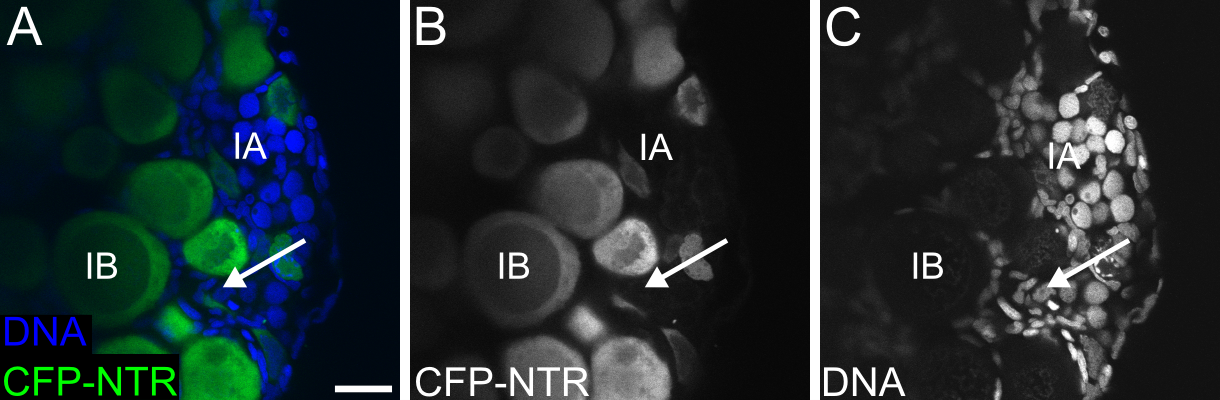

Supplement: S1 Fig — (A-C) Expression of zp3:CFP-NTR in the ovary. (A) Merged image: CFP-NTR in green, DNA in blue. (B) CFP-NTR only. (C) DNA only. IB, stage IB oocyte. Arrow indicates premeiotic germ cell. Scale bar in A (for A-C): 20 μm. (TIF) [file pgen.1006323.s001.tif]

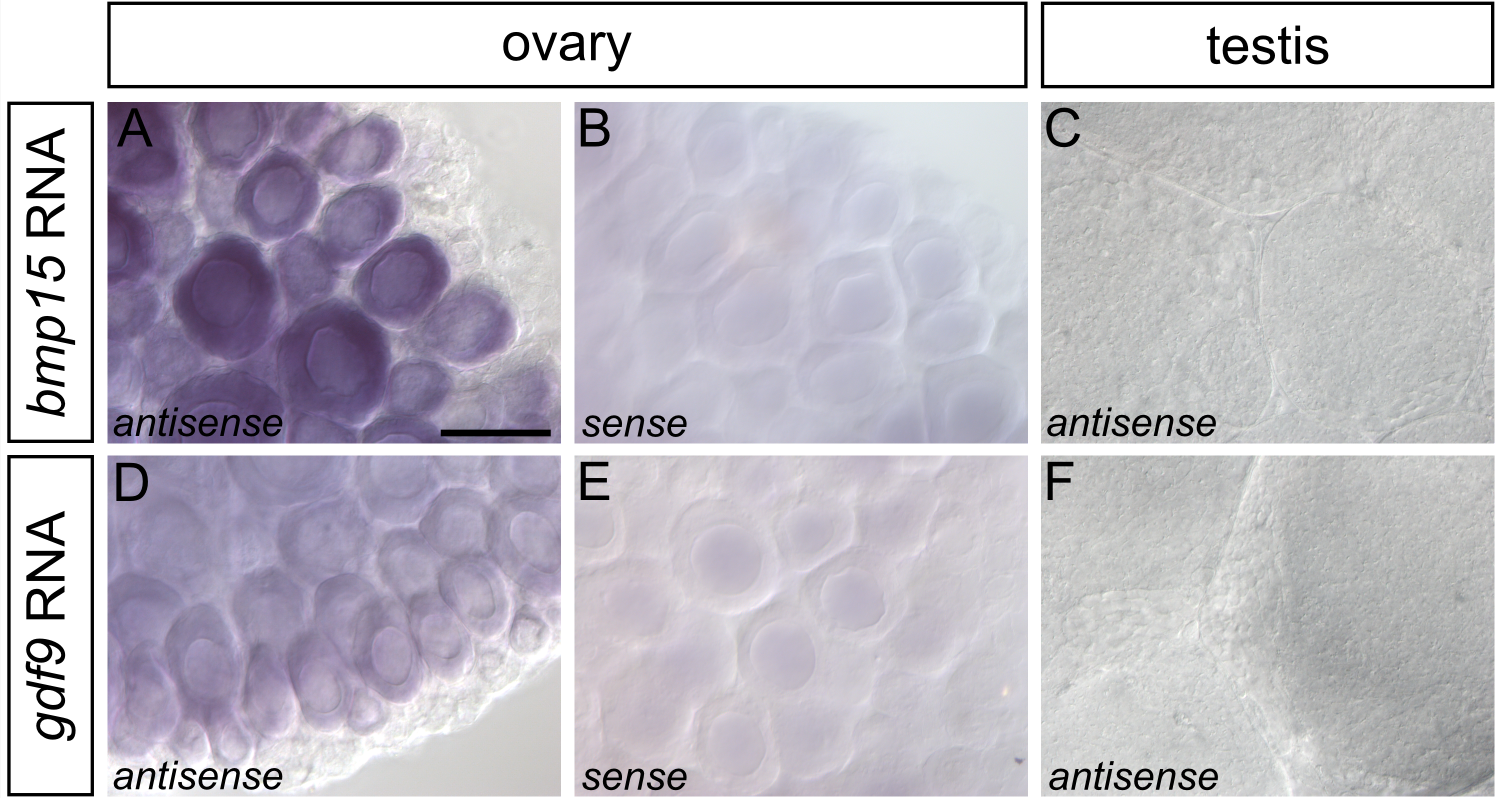

Supplement: S2 Fig — (A-D) In situ hybridization for bmp15 and gdf9 RNA in 40 dpf wild-type gonads. (A) bmp15 and gdf9 (C) RNAs are detected in stage IB oocytes but not the smaller, more immature stage IA oocytes or premeiotic germ cells in ovaries. Neither transcript is detected in wild-type adult testes (B, D). Scale bar in A (for A-F): 50 μm. (TIF) [file pgen.1006323.s002.tif]

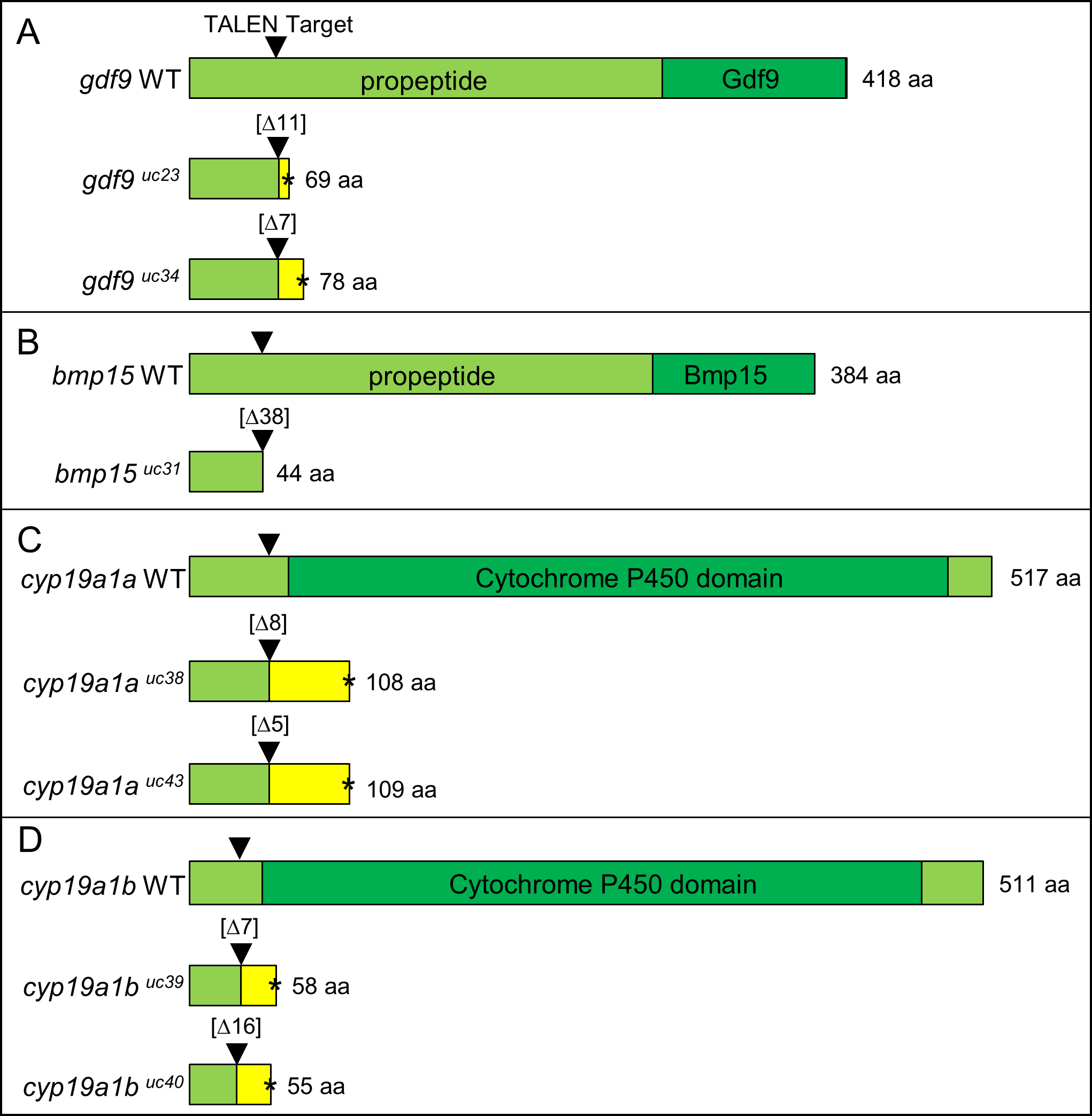

Supplement: S3 Fig — gdf9 (A), bmp15 (B), cyp19a1a (C), and cyp19a1b (D) alleles. Each allele is a predicted loss-of-function mutation. Green indicates wild-type sequence while yellow indicates the predicted frame-shifted sequence. Black triangles indicate TALEN target sites with the size of each deletion bracketed above. Asterisks mark the sites of premature stop codons. The predicted length of the corresponding peptide is indicated on the right. WT, wild type; aa, amino acids. (TIF) [file pgen.1006323.s003.tif]

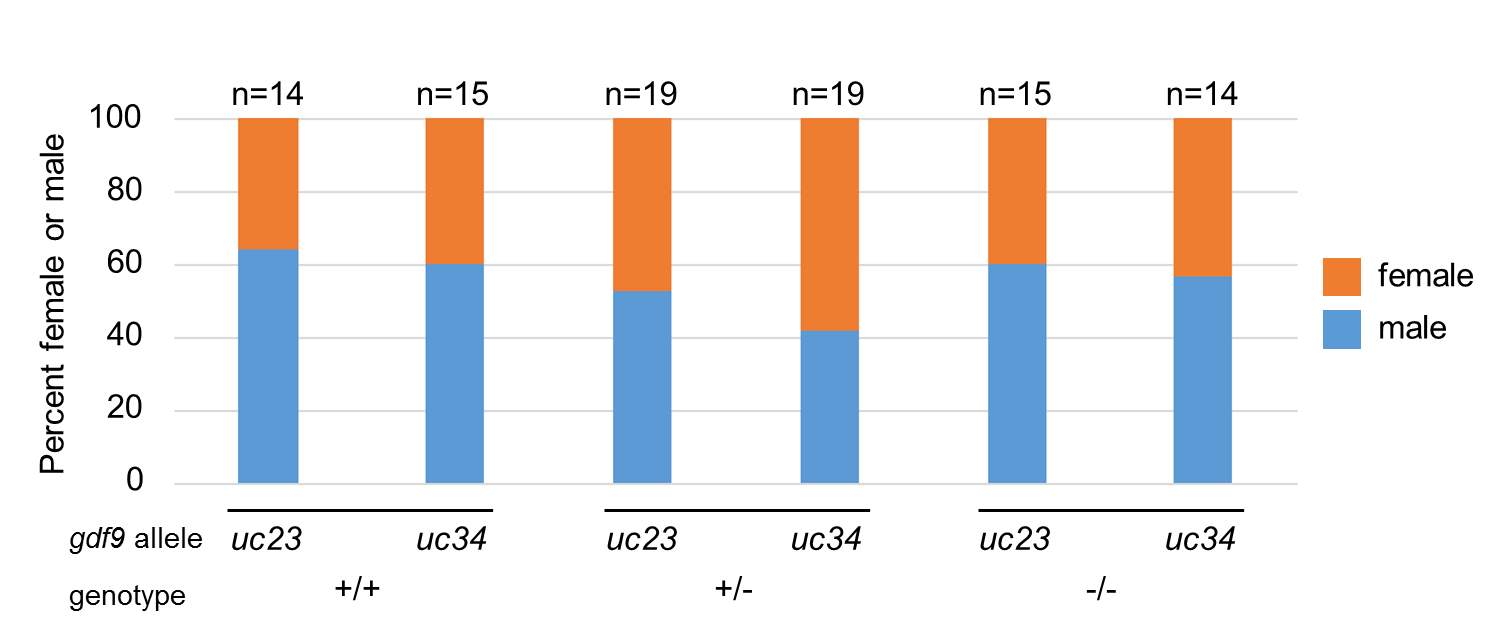

Supplement: S4 Fig — gdf9 mutants (alleles uc23, uc34) were raised to adulthood and their sex determined. (TIF) [file pgen.1006323.s004.tif]

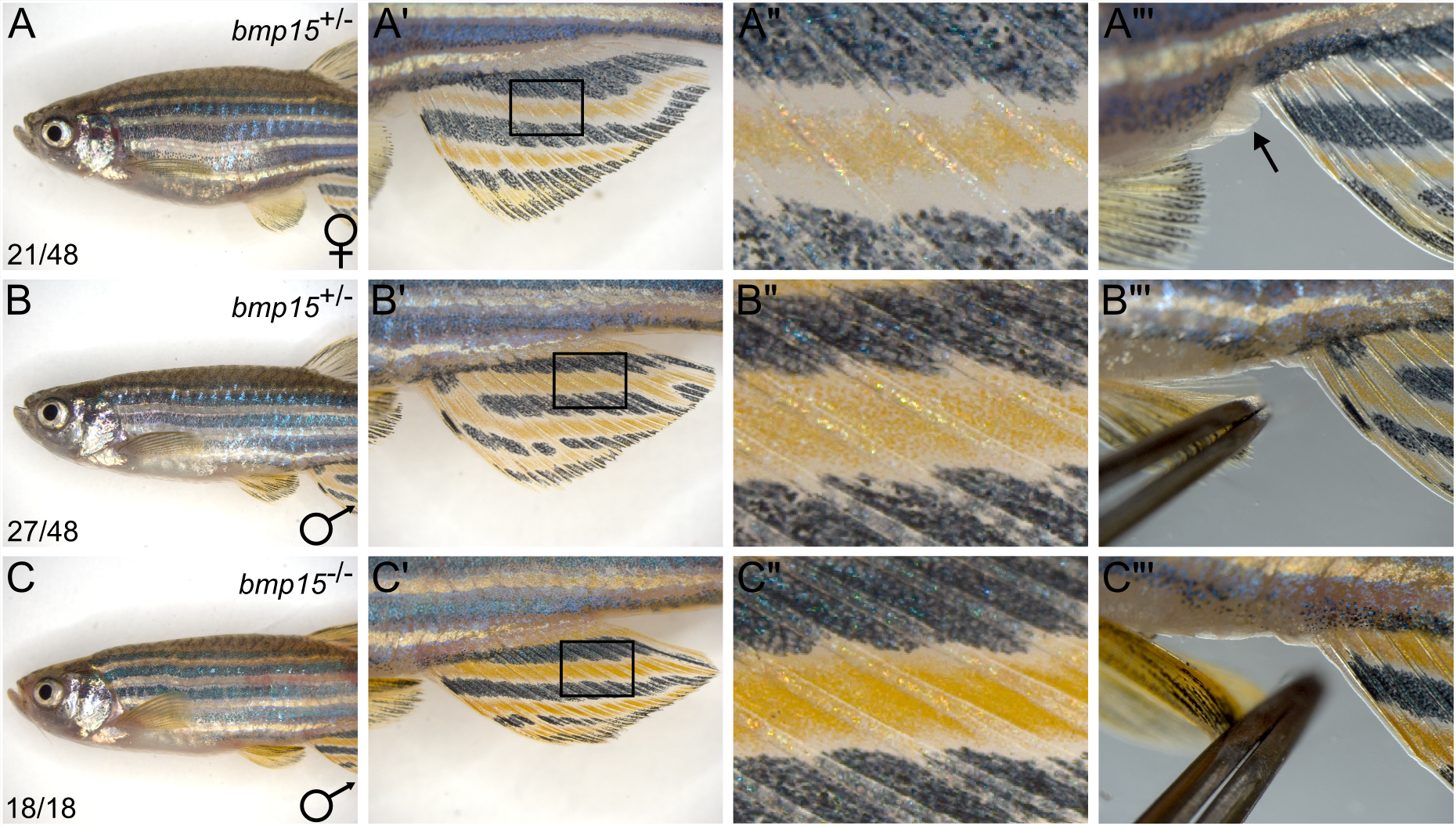

Supplement: S5 Fig — (A-A”’) Adult bmp15+/- female. (B-B”’) Adult bmp15+/- male. (C-C”’) Representative adult bmp15-/- male. (A-C) Anterior body. (A’-C’) Magnified view of the anal fin. (A”-C”) Magnified view of the regions boxed in A’-C’. (A”’-C”’) Magnified view of the cloaca. While females (A”’) have a prominent cloaca (arrow), males (B”’, C”’) do not. (TIF) [file pgen.1006323.s005.tif]

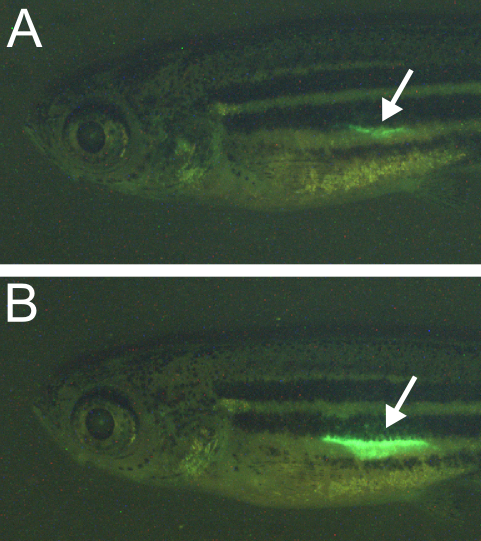

Supplement: S6 Fig — (A, B) EGFP expression in Tg(ziwi:EGFP) fish at 33 dpf. (A) EGFP expression is lowest in presumptive males but greater the larger gonads of presumptive females (B). Arrows indicate the gonad. (TIF) [file pgen.1006323.s006.tif]

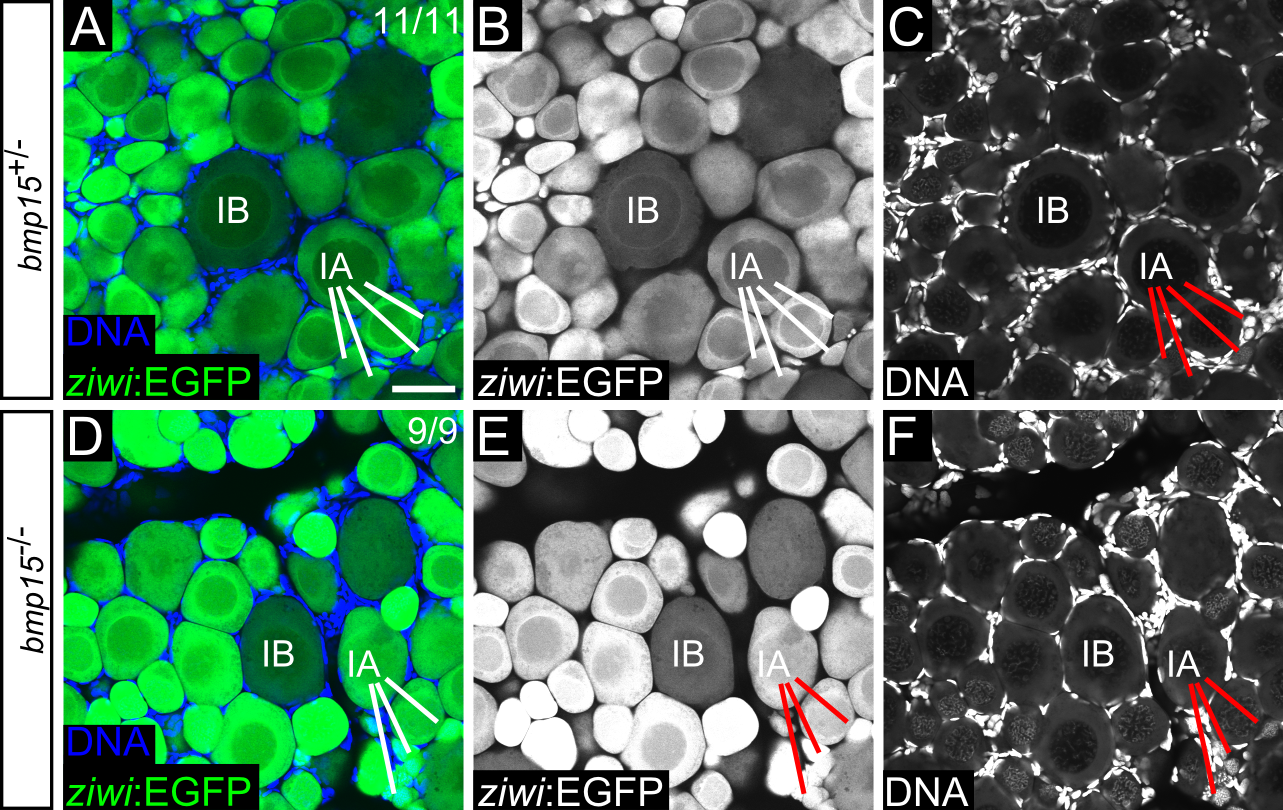

Supplement: S7 Fig — (A-C) bmp15+/- ovaries are indistinguishable from bmp15-/- ovaries (D-F). (A, D) Merged images: ziwi:EGFP in green, DNA in blue. (B, E) ziwi:EGFP only. (C, F) DNA only. IA, stage IA oocytes; IB, stage IB oocytes. Scale bar in A (for A-F): 50 μm. (TIF) [file pgen.1006323.s007.tif]

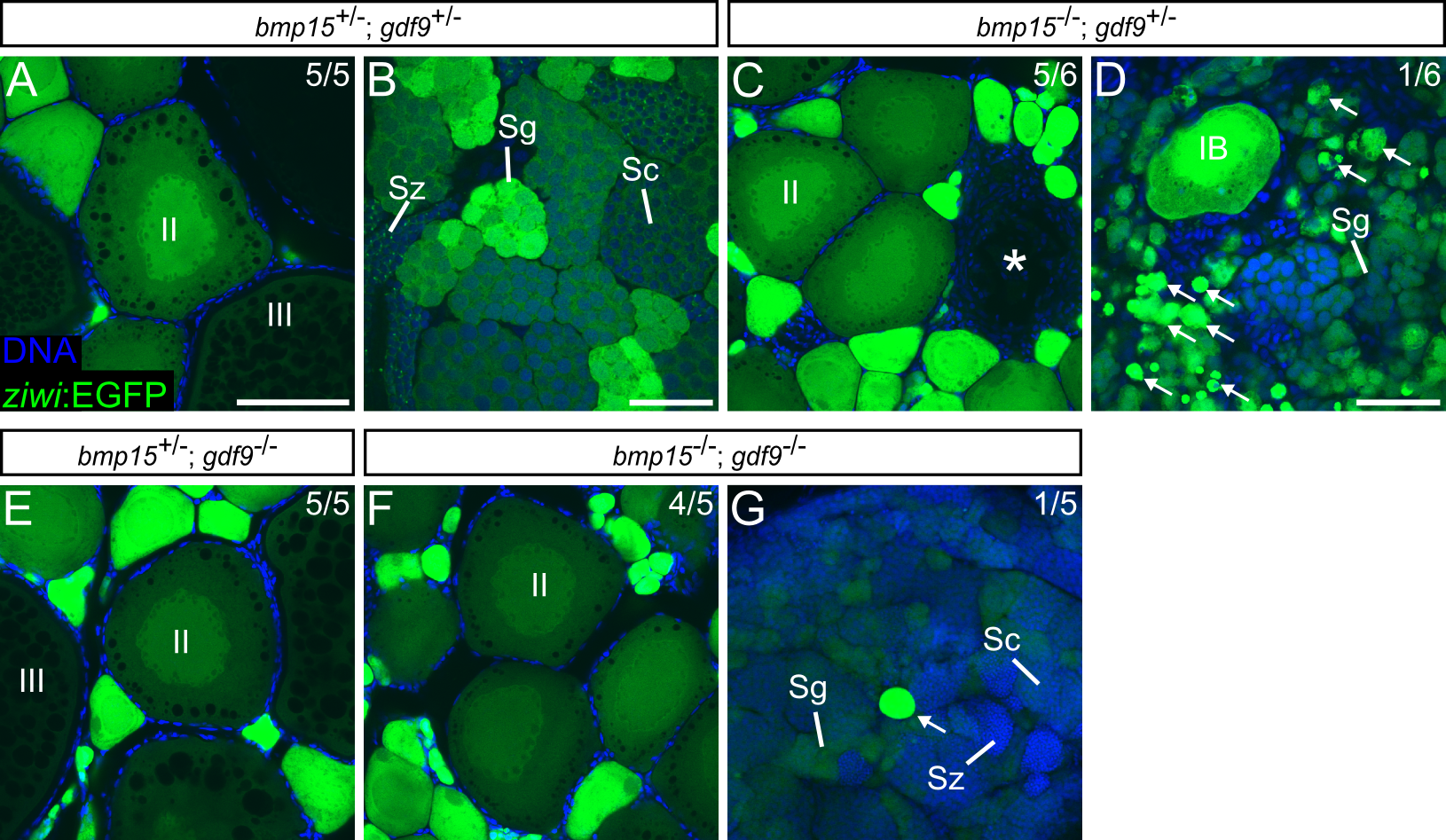

Supplement: S8 Fig — bmp15; gdf9 double mutant analysis (A-G). bmp15+/-; gdf9+/- (A, B), bmp15-/-; gdf9+/- (C, D), bmp15+/-; gdf9-/- (E), bmp15-/-; gdf9-/-(F, G). ziwi:EGFP in green, DNA in blue. Arrows indicate degrading oocytes and asterisk indicates empty follicle. IB, II, III indicate oocyte stages. Sg, spermatogonia; Sc, spermatocytes; Sz, spermatozoa. Scale bars: 100 μm in A (for A, C, E, F and G), 25 μm (B), 50 μm (D). (TIF) [file pgen.1006323.s008.tif]

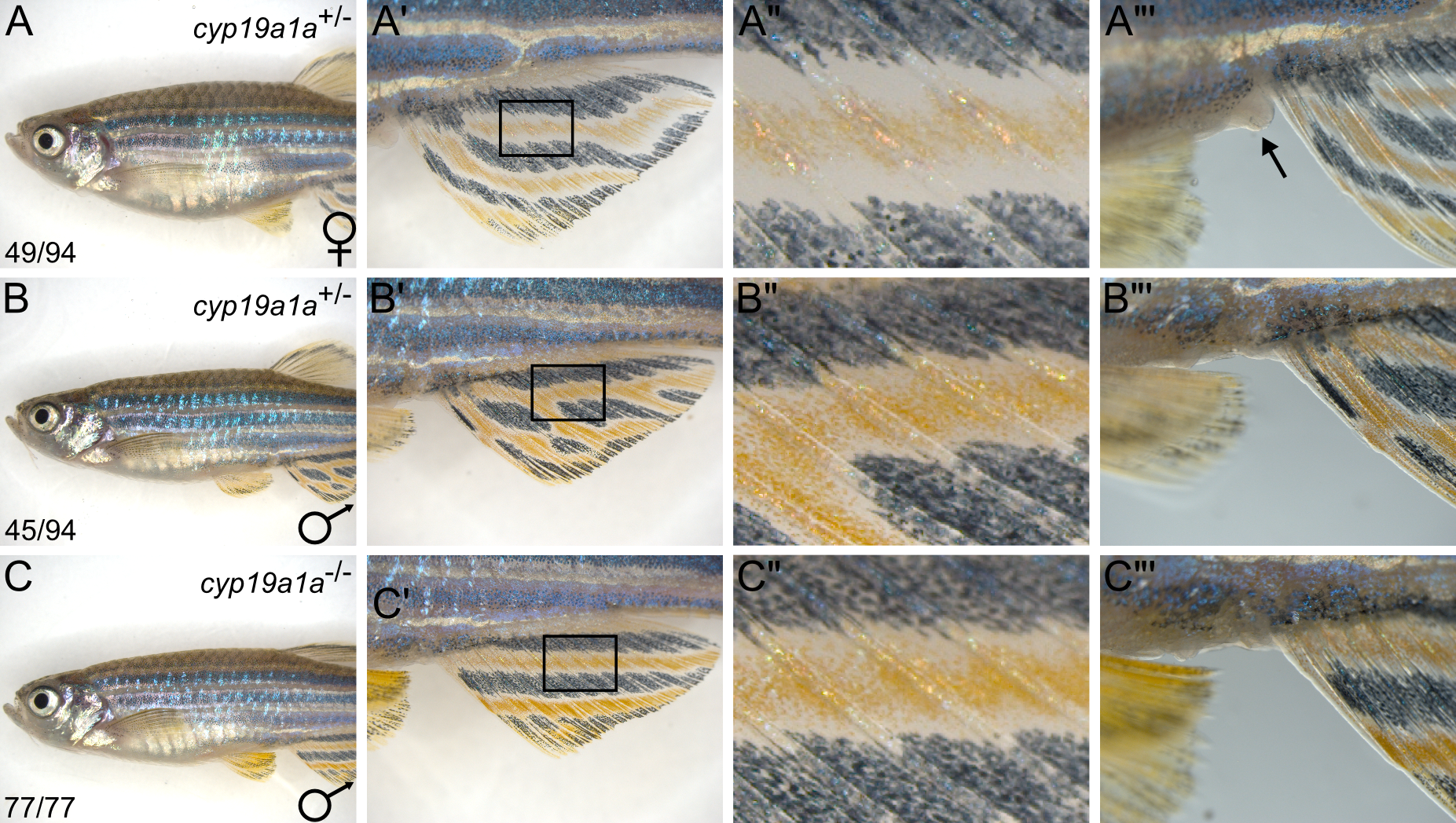

Supplement: S9 Fig — (A-A”‘) Adult cyp19a1a+/- female. (B-B”‘) Adult cyp19a1a+/- male. (C-C”‘) Representative adult cyp19a1a-/- male. (A-C) Anterior body. (A’-C’) Magnified view of the anal fin. (A”-C”) Magnified view of the regions boxed in A’-C’. (A”‘-C’”) Magnified view of the cloaca. While females (A”‘) have a prominent cloaca (arrow), males (B”‘, C”‘) do not. (TIF) [file pgen.1006323.s009.tif]

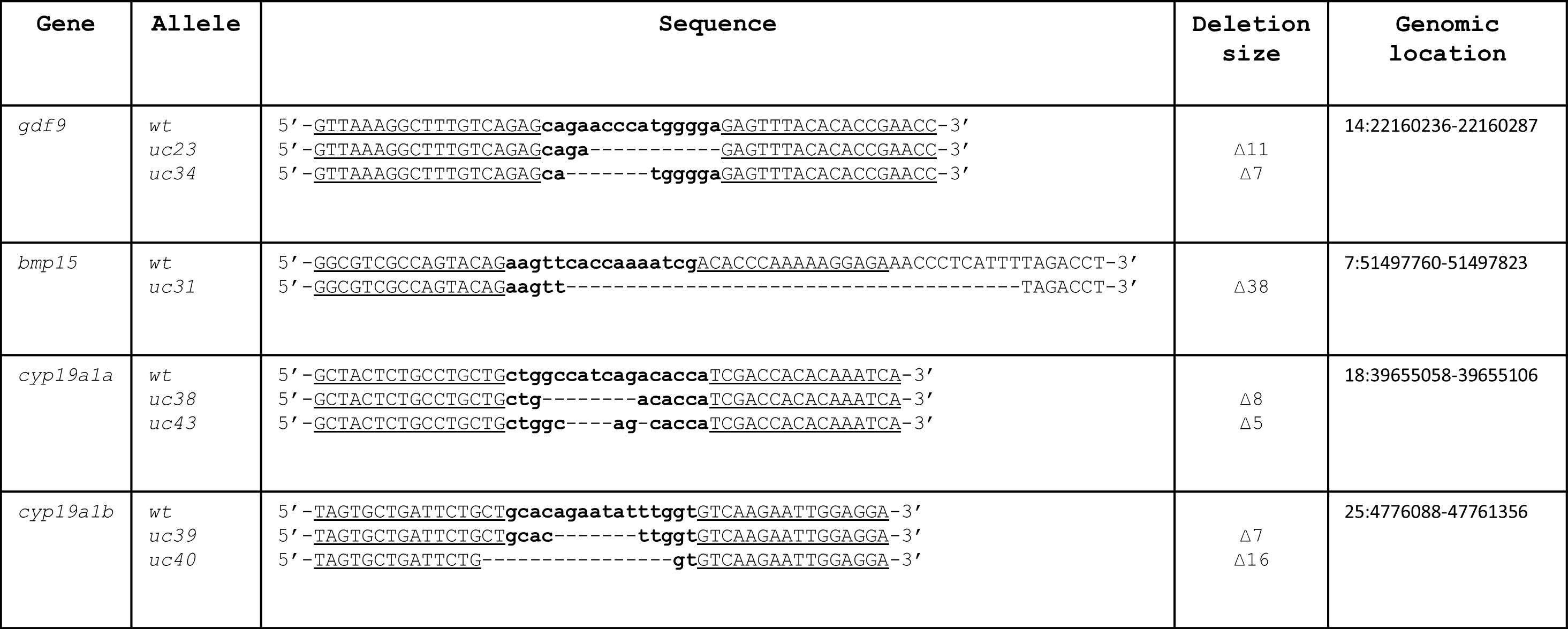

Supplement: S1 Table — Sequence between binding sites is lower case and bold. Sequences are in 5’ to 3’ direction. (TIF) [file pgen.1006323.s010.tif]

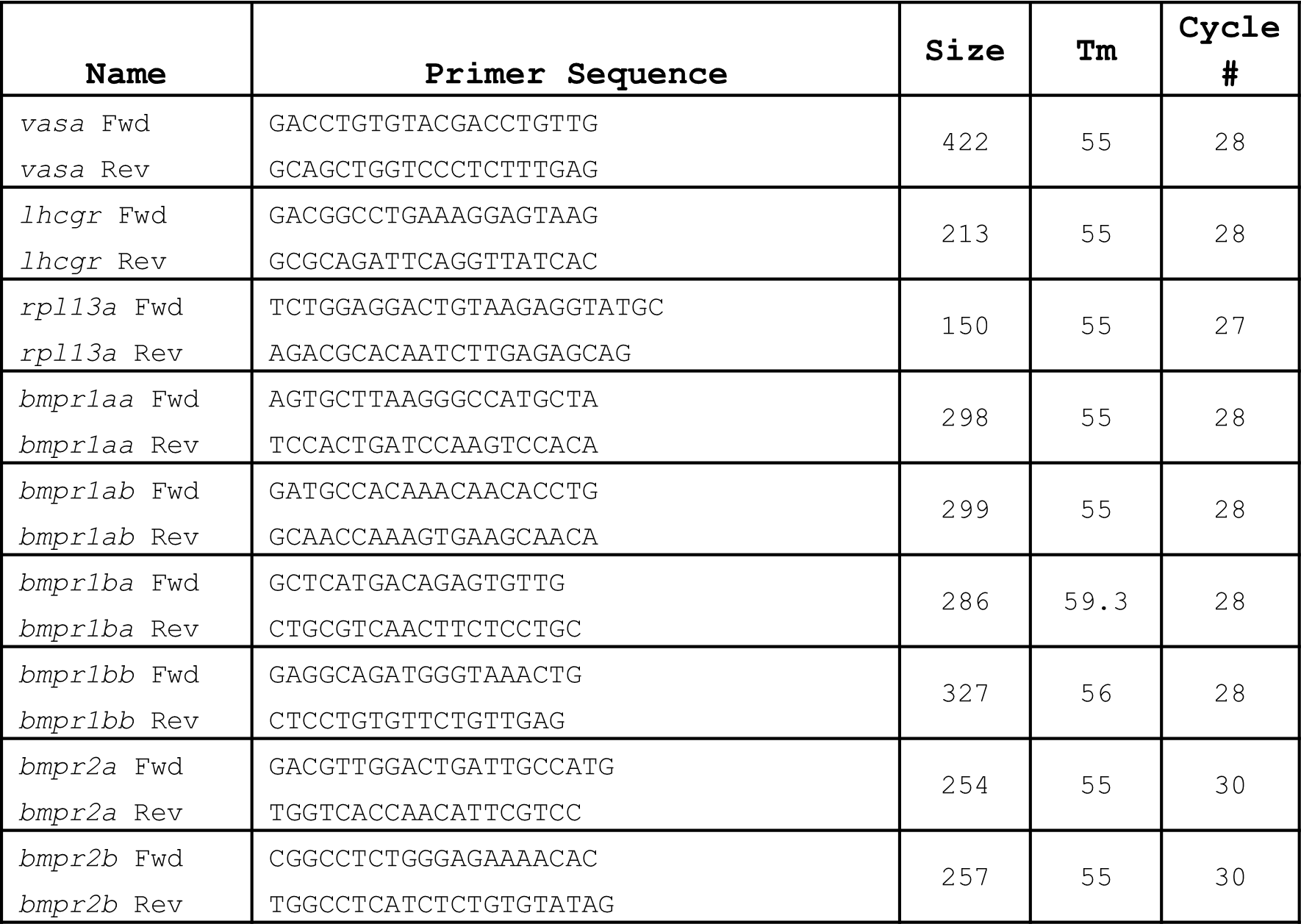

Supplement: S2 Table — The annealing temperature and numbers of PCR cycles used for each gene-specific primer set are listed. (TIF) [file pgen.1006323.s011.tif]
